# Deciphering opening mechanisms of 14‐3‐3 proteins

**DOI:** 10.1002/pro.70108

**Published:** 2025-03-25

**Authors:** Exequiel E. Barrera, Rostislav Skrabana, Diego M. Bustos

**Affiliations:** ^1^ Instituto de Histología y Embriología de Mendoza (IHEM) Universidad Nacional de Cuyo, CONICET Mendoza Argentina; ^2^ Institute of Neuroimmunology Slovak Academy of Sciences Bratislava Slovakia; ^3^ Facultad de Ciencias Exactas y Naturales UNCUYO Mendoza Argentina

**Keywords:** 14‐3‐3, allosteric mechanisms, conformational plasticity, cross‐correlation based tools, molecular dynamics

## Abstract

The 14‐3‐3 proteins are a highly conserved family of regulatory molecules that play crucial roles in various cellular processes. They are known for their ability to bind to phosphorylated serine and threonine residues on target proteins, which allows them to modulate their activity, localization, and stability. In mammals, there are seven known paralogs of 14‐3‐3 proteins, designated as β, ε, ζ, η, σ, τ, and γ. Each paralog has distinct biological functions and tissue distributions, which allow a diverse range of regulatory roles in cellular processes. The conformational plasticity of 14‐3‐3s regulates their interaction with protein partners but has not yet been thoroughly characterized. We investigated this topic by classical molecular dynamics simulations and observed how the γ, ε, and ζ paralogs exhibit different opening rates. A PCA analysis identified the main modes of these opening‐conformational variations. Using correlation‐based tools and simulations with single amino acid substitutions, we have recognized how the amphipathic 14‐3‐3 groove opening is triggered by a distally located aliphatic–π interaction. The identified residues form a partially conserved small cavity between helices H6, H7, and H8, representing a potential paralog‐specific drug site.

## 1 INTRODUCTION

The 14‐3‐3 family of proteins was identified in 1966, after a systematic analysis of calf brain proteins (Moore et al., 1968; Moore & Perez, 1967). Its name reflected the fraction number during chromatographic separation and the position on gel electrophoresis.

In humans and mammals, there are nine isoforms of 14‐3‐3 corresponding to seven distinct paralogs coded by individual genes, because some isoforms are phosphorylated forms of the others (Aitken, 2006; Aitken et al., 1992; Isobe et al., 1991). The sequence of the 14‐3‐3 family is highly conserved both between individual paralogs and between species. 14‐3‐3 proteins exist as homo‐ or hetero‐dimers of ~30 kDa monomers, being usually considered as evolved members of the Tetratrico Peptide Repeat (TPR) superfamily of α‐helical proteins (Obsilova & Obsil, 2022; Zhu et al., 2016). TPR proteins are present in all kingdoms of life except in prokaryotes, where they have not been found until present (Sluchanko & Bustos, 2019a).

Elucidation of the structures of 14‐3‐3 proteins (Liu et al., 1995a; Xiao et al., 1995), and the description of their function as proteins recognizing phosphoserine and phosphothreonine residues in a large variety of protein sequences (Muslin et al., 1996; Yaffe et al., 1997; Yang et al., 2006) significantly increased the interest in this protein family. Decades of research have shown inductively that 14‐3‐3 proteins are involved in many cellular processes via the regulation of phosphorylatable targets through direct interaction. Their targets are classified in a wide range of cellular functions, like metabolic enzymes, transcription factors, signaling and cytoskeleton proteins, apoptotic proteins, and cell cycle components (Aitken, 2006; Mackintosh, 2004; Obsilova et al., 2008; Sluchanko, 2018; Sluchanko & Bustos, 2019a).

Out of the seven identified human 14‐3‐3 paralogs (β, γ, ε, ζ, σ, τ/θ, η), about 30 years ago, the first crystal structures were determined for 14‐3‐3ζ and 14‐3‐3τ/θ (Liu et al., 1995a; Xiao et al., 1995). Analysis of their structures shows that 14‐3‐3 monomers are composed of nine α‐helices (H1–H9) arranged in a simple antiparallel fashion, with tight lateral interactions between neighboring helices. There are also conserved interactions between non‐adjacent helices, contributing to the overall shape and stability of the 14‐3‐3 globule. Two monomers compose W‐shaped dimers via their N‐terminal helices H1–H4, interconnected in an antiparallel way. These N‐terminal helices represent a flat base of the dimer, surrounded by C‐terminal helices H5–H9 in the form of nearly perpendicular walls on both sides of the base.

Each monomer's inner side contains an amphipathic binding groove parallel to the dimer internal edge where 14‐3‐3's targets bind, involving helices H3, H5, H7, and H9. The amino acid sequence of the targets is important for binding efficiency. The optimal consensus sequences for 14‐3‐3 binding are *motif I* ..RXXpSXP.., *motif II* ..RXXXpSX[P/L/M].., and the C‐terminally located *motif III* ..(pSX_1–2_–COOH).., where pS stands for phosphorylated serine (or threonine) and X for any residue (Coblitz et al., 2006; Ganguly et al., 2005; Yaffe et al., 1997). Such sequences can occur once, twice, or more in a given protein, which dictates how it binds to 14‐3‐3. The phosphate recognition occurs mostly by electrostatic interactions in the positively charged pocket formed by the evolutionary restricted residues K49, R56 (in H3), and R127, Y128 (in H5; human 14‐3‐3ζ residue numbering). A C‐terminal hydrophobic cluster in H7 and H9 on the lobe of each monomer complements the positively charged pocket to interact with the phosphopeptide backbone (Liu et al., 1995a; Obsilova & Obsil, 2022; Yaffe et al., 1997). The amphipathic groove of the 14‐3‐3 dimer is highly conserved (Liu et al., 1995a) and is the structure that supports the phosphopeptide recognition mechanism along the protein family. In contrast, the outer surface of the 14‐3‐3 dimer is much more evolutionarily variable, and no biologically relevant sites for 14‐3‐3 function have been discovered to date.

Accessibility of the binding groove defined by the mutual position of the dimer base and dimer walls may substantially influence the kinetics of phosphopeptide recognition. Although early crystal structures suggested almost invariant conformations of the 14‐3‐3 dimer, later evidence indicated that the degree of openness of the C‐terminal lobe relative to the dimer base may differ significantly (Yang et al., 2006; Nagy et al., 2017; Brokx et al., 2011; Sluchanko et al., 2017), and this transition between closed‐to‐open states was suspected to be crucial in target recognition and binding (Nagy et al., 2017; Sluchanko et al., 2017; Hu et al., 2014).

Here, we investigated the conformational plasticity of three 14‐3‐3 paralogs, the transient opening and closing conformations, and the importance of distant residues in the dynamics of 14‐3‐3 proteins. Our results suggest an allosteric site at the outer side of the 14‐3‐3 dimer that may be coupled with the closing/opening of the binding groove.

## 2 RESULTS

### 2.1 Opening‐closing dynamics measurement of paralogs γ, ζ, and ε

**FIGURE 1:**
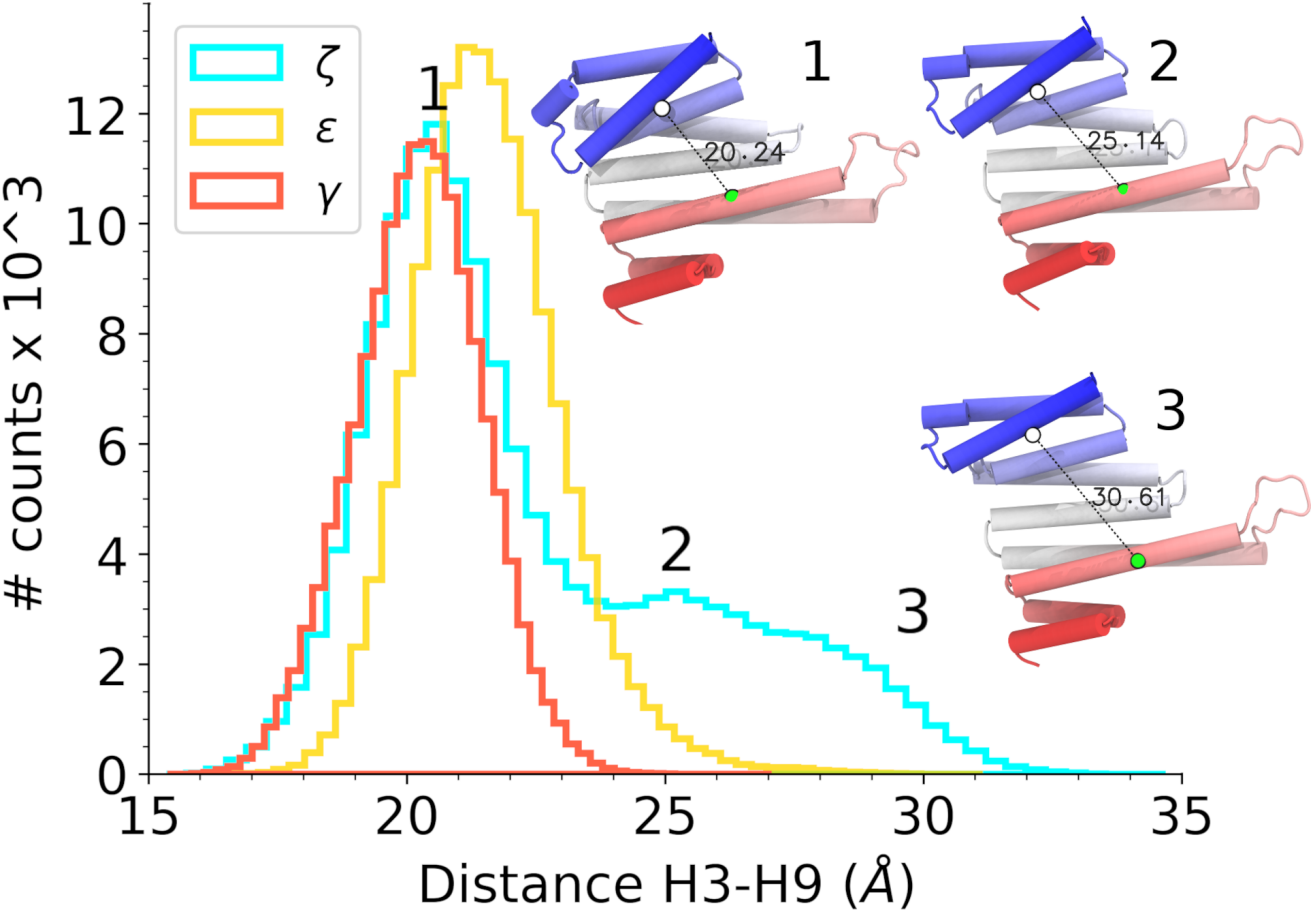
Histogram representation of γ, ζ, and ε 14‐3‐3's amphipathic groove opening. On the right, cartoon representations of different representative conformations of the ζ paralog corresponding to distribution peaks 1, 2, and the distribution's tail are shown. Distances were measured between Cα atoms of residues G53 and L220 (ζ residue numbering), which are displayed with green and white van der Waals representations, respectively.

We studied the conformational plasticity of 14‐3‐3 paralogs γ, ζ, and ε by performing molecular dynamics (MD) simulations of monomeric apo species in explicit solvent. Upon visual inspection of their trajectories, we observed transient opening and closing events within the amphipathic groove. To quantify these events, we measured distances between the Cα atoms of G53 and L220 (ζ‐paralog), G52 and L221 (ε paralog), and G53 and L224 (γ paralog) located at the centers of helices H3 and H9, respectively. These helices encompass the edges of the amphipathic groove. Distance distributions for the three 1 μs simulated replicas are depicted in Figure 1. Here, we observed how the γ‐paralog is characterized by a narrow H3–H9 distance distribution ($\bar{x}=20.39$ Å and ±2σ ranges between 17.94 and 22.84 Å). For the case of ε‐paralog, the population is shifted to slightly more open conformations (21.3 Å and ±2σ between 18.39 and 24.21 Å). Differently, 14‐3‐3ζ has the highest conformational plasticity with a trimodal distance distribution presenting peaks at 20.5, 25.2 Å, and a tail of the distribution surpassing distances of 30 Å.

### 2.2 Correlation‐based analysis unveils distal regions controlling 14‐3‐3's conformational plasticity

**FIGURE 2:**
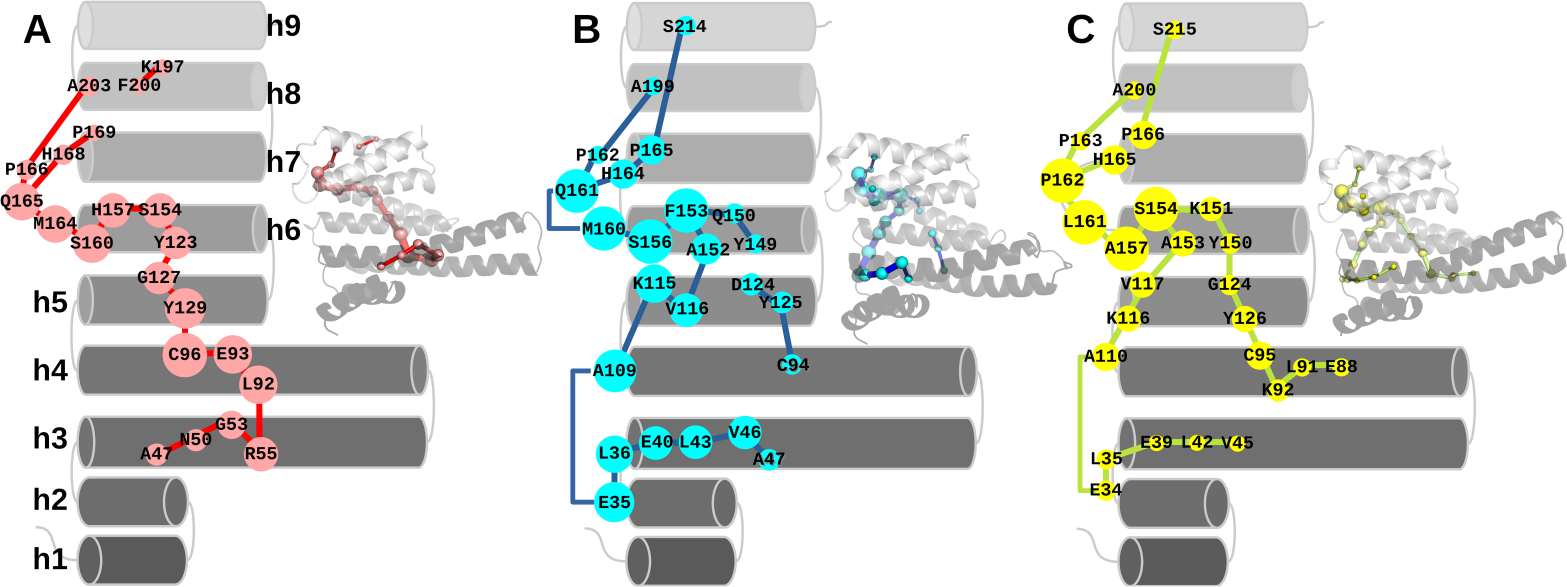
Residues mostly involved in 14‐3‐3's conformational plasticity. 2D scheme showing the shortest path maps for paralogs γ, ζ, and ε (panels A, B, and C, respectively). Interconnected nodes are represented in colored spheres, and the corresponding residues are displayed with one‐letter code residue naming. These residues present the highest displacement correlation through the 3 μs accumulated simulation time. A 3D representation is also shown.

Key residues that regulate the conformational landscape of proteins can be identified by analyzing MD trajectories with correlation‐based tools (Duran et al., 2024). One of these, DynaComm (Casadevall et al., 2024) was employed to decipher the mechanisms controlling the plasticity of each paralog. This method, based on graph theory, represents each Cα of the protein with a node, connecting them with an edge when their distances across the full trajectory are shorter than a certain cutoff distance. We first employed a value of 6 Å, as previously reported (Duran et al., 2024; Osuna, 2021). The displacement correlation between Cα atoms will be directly proportional to the node sizes, reducing inter‐node distances. Finally, the Dijkstra algorithm is applied to traverse all the nodes, selecting those that are more closely connected and generating the shortest path map (SPM) (Figure 2).

The analysis of the generated maps for the three paralogs revealed different correlations for residues belonging to helices H2–H5 but also showed similarities, particularly in the high correlations observed between residues from helix H6 and the loop that connects it to H7. These are located on the 14‐3‐3 dimer outer side and do not include residues of the amphipathic groove that could interact with protein partners. The shortest path for γ runs through the central portions of helices H3–H5, different from ζ, whose path is displaced towards the loops connecting helices H2–H3 and H4–H5. Once again, ε paralog showed an intermediate behavior, presenting two well‐defined branches. One of them is similar to γ, located at the helices' centers, whereas the other runs through the same loops as ζ.

The amphipathic groove is highly conserved between the seven human paralogs (Uhart & Bustos, 2013) (Figure S1), and cannot account for the conformational differences observed in our simulations. Thus, guided by the SPMs, we focused on helices H6, H7, and H8; and observed a previously undescribed small cavity formed by residues that are not fully conserved across paralogs (Figure S2). Using the VolSite method (Tran‐Nguyen et al., 2019), included in the OpenEye software package (Molecular Modeling Software, n.d.), we measured the volumes (vol) and estimated the druggability (drg) of this cavity from the most populated cluster in each studied paralog. Details on the parameters employed to predict the druggability are included in the materials and methods sections. 14‐3‐3ζ's cavity was characterized by a vol = 564 Å^3^, drg = 0.77; γ: vol = 469 Å^3^, drg = 0.4; and ε: vol = 597 Å^3^, drg = 1.14 (positive drg values correspond to druggable cavities). The less conserved residue of the cavity corresponds to F153 (ζ), H157 (γ), and S154 (ε) in H6, which is partially exposed to the solvent and located at the entrance of the cavity of each paralog. Additionally, we performed an amino acid conservation analysis within individual paralogs across more than 500 14‐3‐3 orthologs, for each residue forming the cavity (Figure S3). As shown, most of the cavity residues are highly conserved between species, except for the case of the ε‐paralog, where position 153 (ζ‐paralog numbering) was the most variable with a degree of preservation of 90.9%. These results agree well with the analysis of amino acid conservation between paralogs (Figure S2a).

**FIGURE 3:**
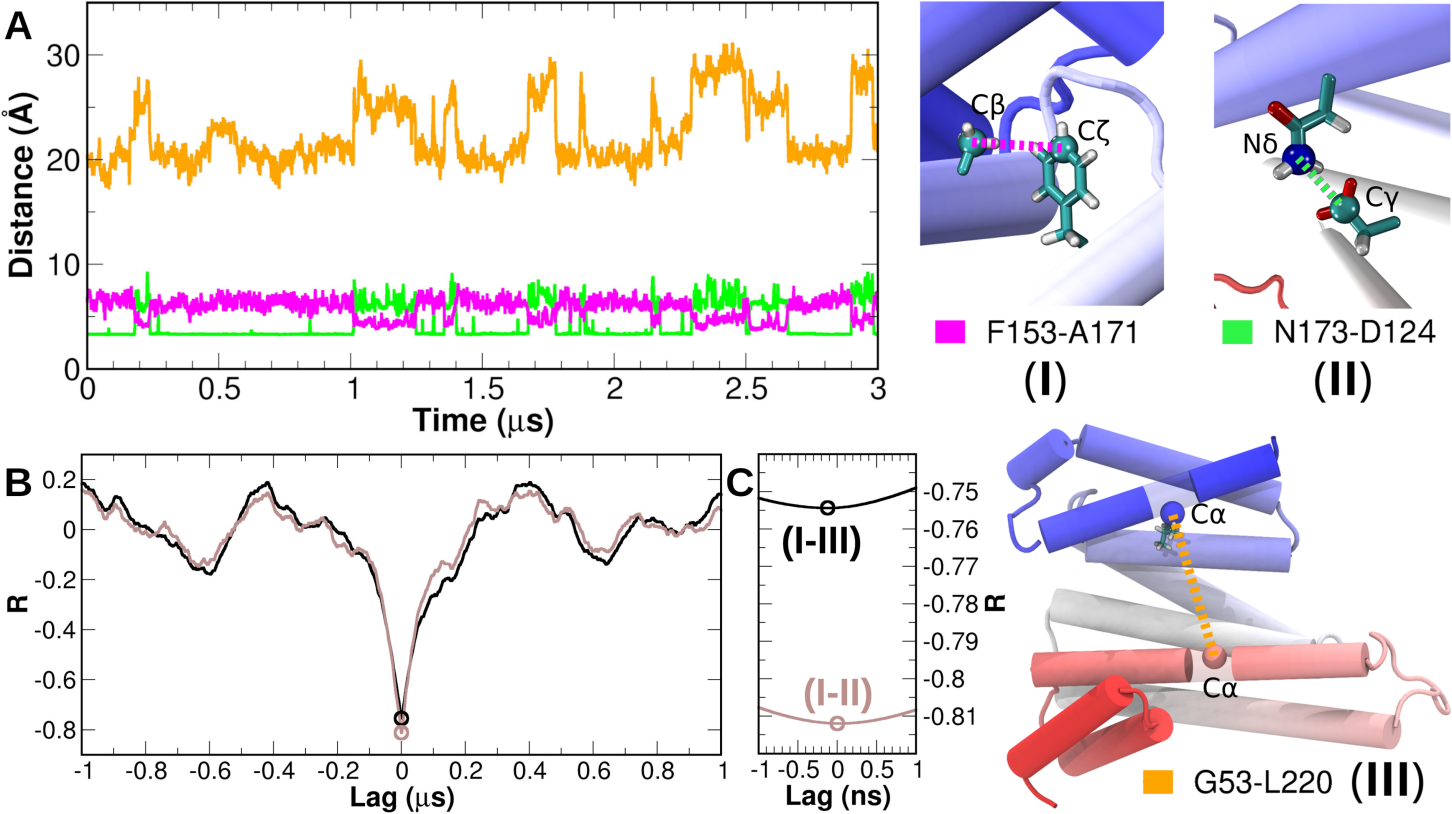
Analysis of the MD trajectories for 14–3‐3ζ. (a) Concatenated time series distances from the three simulated replicas of 14‐3‐3ζ. Distances between F153‐A171 (I, aliphatic–π interaction) and N173–D214 (II, hydrogen bond) are colored pink and green, respectively. In orange, the amphipathic groove opening, measured between G53 and L220 (III). Atoms chosen for the measurements are depicted in the right panel. (b) Cross‐correlation functions between distances I–II (brown) and I–III (black). (c) Zoom in on the lowest *R* values, highlighting the lag time between events.

We inspected the behavior of the cavity residues over the simulated trajectories and observed a transient aliphatic–π interaction between F153 and A171 in helices H6 and H7, respectively, of ζ‐paralog. To calculate a possible correlation between this interaction and the opening of the amphipathic groove, we employed a cross‐correlation function to analyze the time series of the distances between the side chains of residues F153 and A171 (Cζ and Cβ atoms, respectively) and those used to measure the opening of the amphipathic groove (G53‐L220 Cα atoms). Results showed the highest anti‐correlation between events at *R* values of −0.75 and negative lag times of 0.15 ns, indicating that the aliphatic–π interaction precedes the opening of the amphipathic groove (Figure 3b,c). We also detected fluctuating hydrogen bond interactions between residues N173 and D124 interconnecting helices H5 and H7. We measured distances, this time between N173 Nδ and D124 Cγ atoms, and calculated their cross‐correlation with the F153–A171 distances as before. The highest anti‐correlation of *R* = −0.81 was observed at a lag time of 0 ns, suggesting that the F153–A171 aliphatic–π interaction and the break of N173–D124 H‐bond occur simultaneously, with the consequent amphipathic groove opening. These events were reversible, observing the opposite succession as the groove gets closed (Figure 3a).

### 2.3 Rational mutant modeling to alter 14‐3‐3's conformational space

**FIGURE 4:**
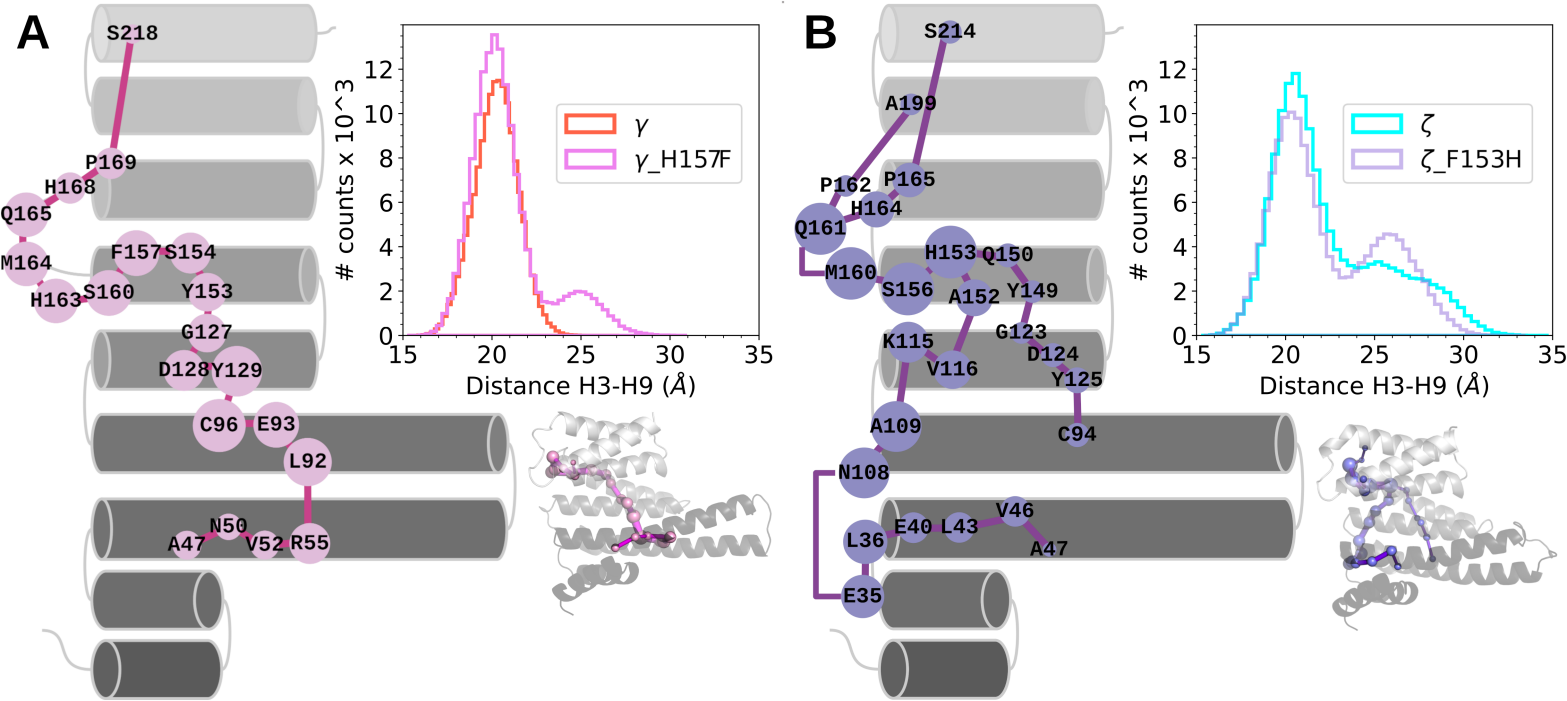
SPM and conformational analysis for (a) γ_H157F and (b) ζ_F153H mutants. 2D schemes SPM for the paralog‐mimetic mutants are shown. The 3D SPM representations are also shown here. A comparison of inter‐helix H3–H9 distance distributions between mutants and wild‐type species is located in the upper right corner of each panel. Distances were measured using the same pair of Cα atoms as in the wild‐type species.

To evaluate our hypothesis about the aliphatic–π interaction relevance on the opening of 14‐3‐3, we generated two paralog‐mimetic mutants switching the non‐conserved residue from the small cavity between γ and ζ (γ_H157F and ζ_F153H mutations) and subsequently simulated trajectories of 1 μs by triplicate (Figure S4). The analysis of inter‐helix H3–H9 distances for γ_H157F indicated that this single mutation was sufficient to modify the distance distribution of the wild‐type paralog, with the appearance of open conformers represented by a secondary peak at 25 Å (Figure 4a). Regarding the ζ_F153H mutant, simulations showed decreased populations of wide‐open conformations, with the loss of the 30 Å tail, translating into an increase in the 25 Å peak (Figure 4b). Unexpectedly, SPM analysis for both mutants showed a high similarity to their wild‐type versions. To evaluate the displacement correlation between more distant residues, we increased the cutoff distance up to 8 Å and observed how γ_WT presented a high correlation between residues D128 and N177 and how these nodes decreased their sizes for the γ_H157F mutant. A high correlation between the residues that stabilized closed conformations was found for ε_WT, while being absent for ζ_WT (Figure S5). It is worth mentioning that even though SPMs and inter‐helix distance analysis were modified with these single mutations, they did not result in a complete mimicking behavior; therefore, implications of other non‐conserved residues over 14‐3‐3's conformational landscape should not be discarded.

To better identify the dominant motions hidden behind the conformational plasticity of our 14‐3‐3 models, we performed principal component analysis (PCA) over each simulated system. Once again, we observed different behaviors for each paralog. A plot of the top 10 eigenvectors' eigenvalues presented the same trend between paralogs as observed in their opening rates (ζ_WT > ζ_F153H > γ_H157F > ε_WT > γ_WT) (Figure S6a). Plotting the cumulative variance versus the top 20 PCs (Figure S6b) indicated that the first two PCs captured 50% of conformational changes observed for ζ_WT, ζ_F153H, and γ_H157F, the systems characterized by significant opening rates. Therefore, we examined the first two principal components from ζ‐paralog's trajectories and observed how the largest atomic displacements corresponded to the opening and closing of the amphipathic groove. By interpolating the extreme conformations along these two eigenvectors, we observed the oscillating positions between helices H3–H9 and how helices H5–H6 acted as a hinge, presenting a rigid behavior (Figure S7). On the opposite side, γ‐paralog's dominant motions mainly correspond to the flapping behavior of the end of helix H3, the beginning of H4, and its connecting turn, with reduced movements of H9. The inclusion of a single point mutation on γ_H157F conferred larger flexibility to helices H3 and H9, in a similar fashion as for ζ_WT. To exclude the possibility that the rigid behavior of γ‐paralog was related to insufficient sampling of the conformational space, we calculated the cosine contents associated with the first 5 PCs. Cosine values close to one correspond to trajectories that have not yet converged and should be run longer (David & Jacobs, 2013). Performing this analysis over the concatenated trajectories for γ‐paralog, we obtained low cosine values (PC1 = 0.00013, PC2 = 0.00869, PC3 = 0.00670, PC4 = 0.01865, and PC5 = 0.01268), confirming that trajectories converged. The convergence of trajectories was confirmed also for the rest of the simulated systems (Table S1).

**FIGURE 5:**
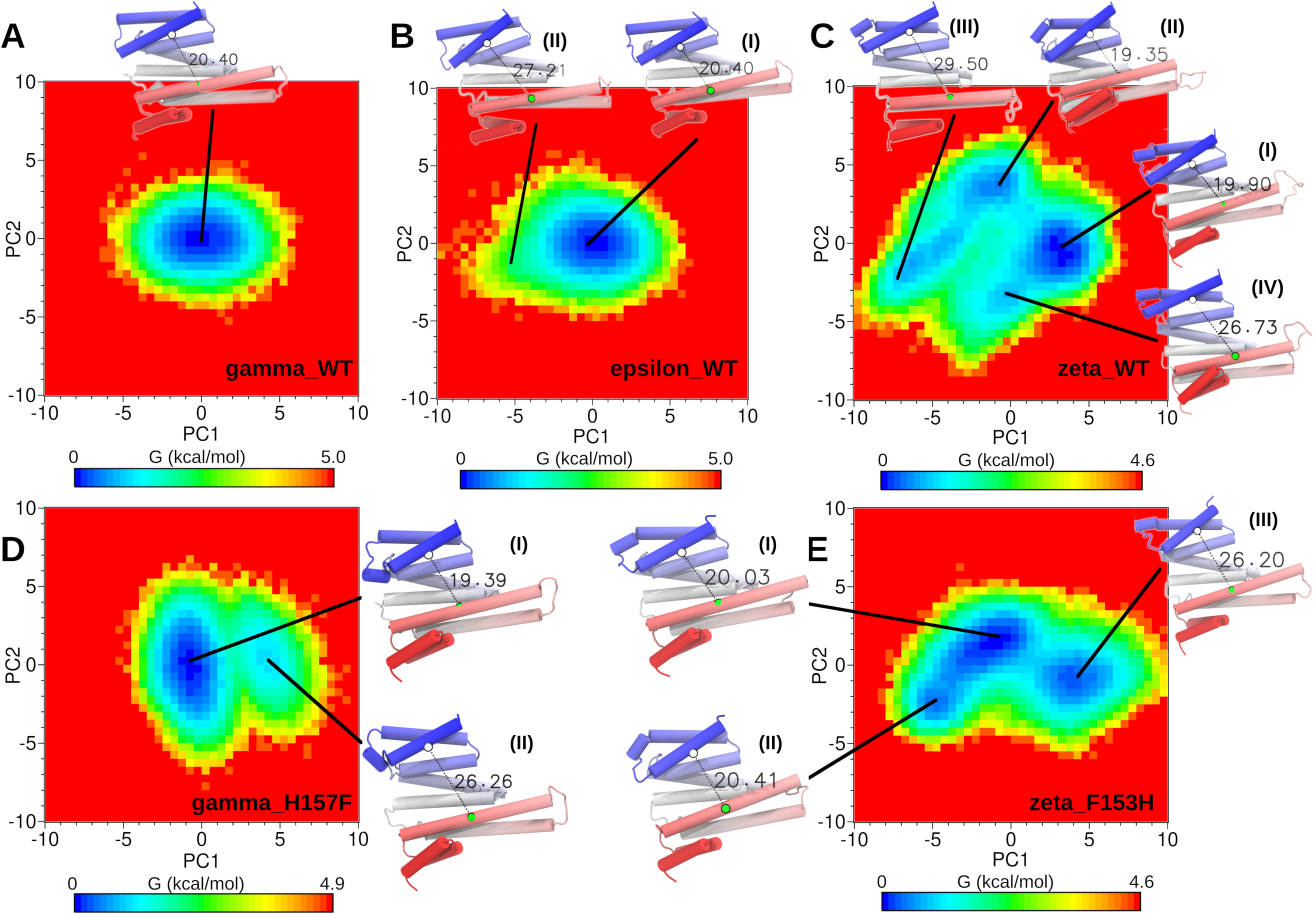
Free energy landscapes (in kJ/mol) along the first two principal components of (a) γ_WT, (b) ε_WT, (c) ζ_WT, (d) γ_H157F, and (e) ζ_F153H systems. Representative structures from each global and local minimum are shown in cartoons, displaying inter‐helix H3–H9 distances.

To identify conformational paths connecting different energy minima, we generated a free energy landscape (FEL), performing a Boltzmann inversion of 2D histograms from the two first principal components (Figure 5). Once again, we started our analysis with the more flexible ζ‐paralog and identified four energy minima. Two of them corresponded to closed states, one leading to open conformations corresponding to the second peak of the distance distribution (26.7 Å) and the other to a member of the tail of the distribution with a wide‐open conformation (~29.5 Å). As expected, γ_WT presented a single minimum, with a symmetric distribution around it. Similarly, ε_WT also showed a single minimum, this time with a slight landscape deformation, corresponding to low‐populated open conformations. Single point mutations of the residues gating the small cavity translated into the loss of the wide‐open conformation minima for ζ_F153H and the gain of an open conformation minimum for γ_H157F.

Additionally, we analyzed the overall dynamics of the studied 14‐3‐3 paralogs by measuring their root mean square deviations (RMSD) and fluctuations (RMSF) through the 3 μs accumulated trajectories. By overlapping the inter‐helix H3–H9 distances and RMSD time series, we observed how the fluctuation of RMSD values accompanied the opening and closing events (Figure S4). The measure of flexibility by residue obtained with the RMSF analysis indicated that the highest values for each studied paralog corresponded to the H3‐turn‐H4, followed by the H7‐turn–H8‐turn–H9 regions (Figure S8), implicated in the opening and closing of the amphipathic groove. Conformational transitions did not imply significant changes in secondary structure contents (Figure S9).

**FIGURE 6:**
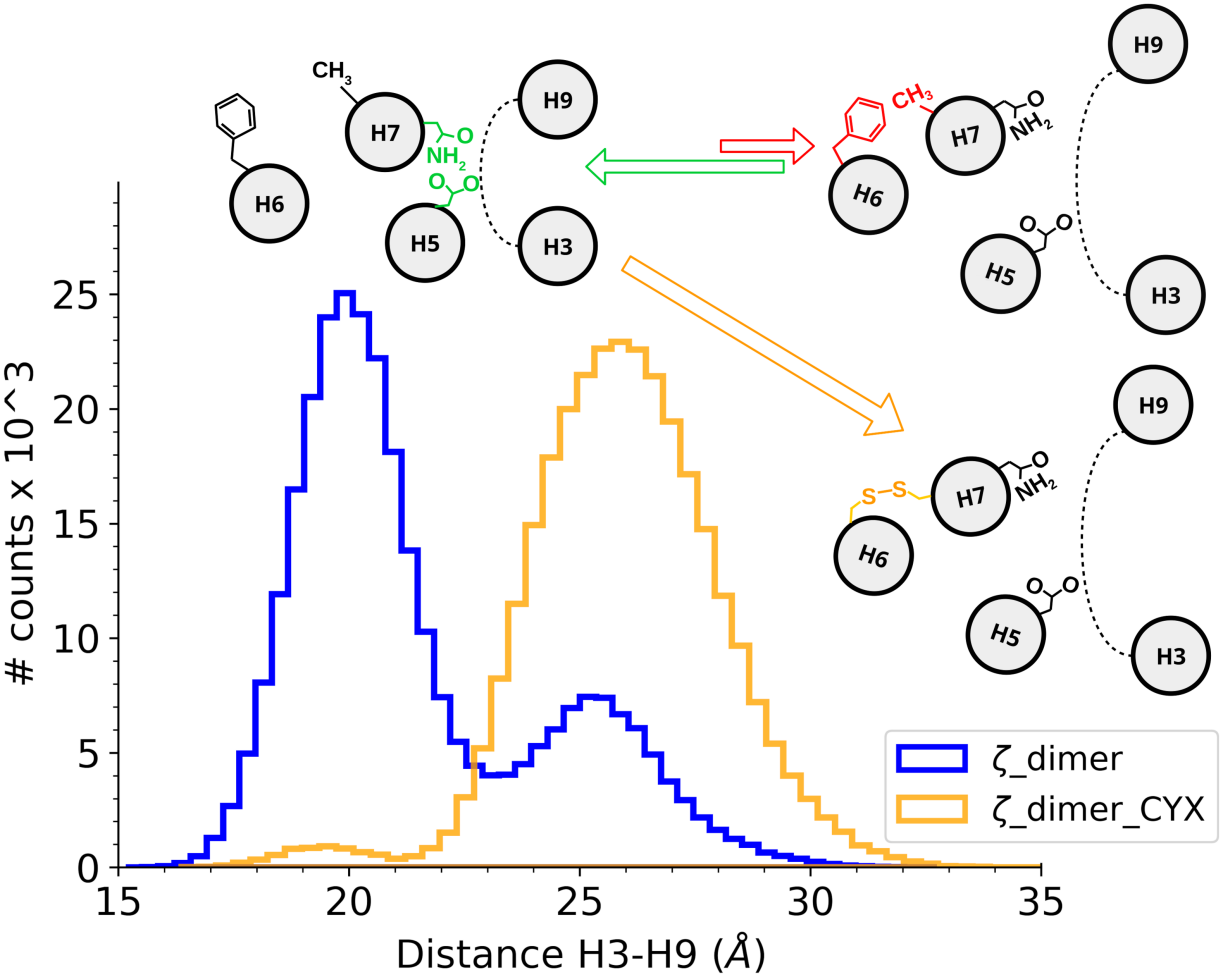
MD analysis of the dimeric 14‐3‐3ζ wild type and cysteine mutants. Inter‐helix H3–H9 distance distributions corresponding to the amphipathic groove opening show a switch to the open conformation after fixing amino acid 153–171 interaction in cysteine mutants. The proposed allosteric mechanism is shown in the scheme. The side view of 14‐3‐3's helices involved in the mechanism is represented with gray circles.

The monomeric forms of 14‐3‐3 coexist in equilibrium with homo‐ and hetero dimers (Trošanová et al., 2022) conserving their ability to bind phosphorylated partners (Sluchanko et al., 2011). The smaller box sizes needed to perform MD simulations make monomeric forms attractive objects of study. To complement the results obtained with monomeric forms, we also included dimeric forms in our work, since they are the predominant biological species. This time, we focused on the more flexible ζ‐paralog. Over the 3 μs accumulated simulation time, dimers showed uncorrelated open conformations of 25 Å for both subunits, but in a less populated way than in the monomeric simulations not presenting the 30 Å tail (Figure 6). Still, the cross‐correlation analysis showed how both D124–N173 hydrogen bond disruption and F153–A171 aliphatic–π interactions control the opening of the amphipathic groove, as observed in the monomeric form (Figure S10). We validated our allosteric hypothesis by designing a double mutant F153C/A171C and covalently joining them with a disulfide bond. The objective of such modification was to freeze the labile interaction between F153 and A171 at the small cavity and observe its effect on opening events. Inter‐helix distance analysis resulted as expected, showing a major peak at 26 Å and presenting only a few counts of the 19 Å closed conformation, corresponding mostly to the beginnings of each simulated replica (Figures 6, S11). The disulfide bond facilitated the break of the hydrogen bond between D124 and N173, irreversibly opening the amphipathic groove.

## 3 DISCUSSION

The flexibility of 14‐3‐3 is a topic that has already been addressed, both experimentally and computationally. Obsil et al. obtained the X‐ray structure of the 14‐3‐3ζ:serotonin N‐Acetyltransferase complex (PDB:1IB1), observing the opening of 14‐3‐3 by a rigid body rotation of their monomers (Obsil et al., 2001). Conversely, Elkins et al. reported the opening of the β‐paralog in its apo form (PDB:2BQ0), this time by conformational changes in one of the monomers' amphipathic grooves (Yang et al., 2006). Moving to the MD field, Wang et al. simulated for brief periods (80 ns) a target‐bound and apo 14‐3‐3σ systems, observing increased flexibility in the latter (Hu et al., 2014). Hritz et al. reported “wide‐open” conformations when studying the interaction pathways of phosphorylated partners by combining Hamiltonian replica exchange MD and distance field restraints (Nagy et al., 2017).

Our study approached for the first time the differential conformational behavior of different 14‐3‐3 variants, focusing on paralogs ζ, γ, and ε. Total simulation times of 3 μs for each paralog allowed us to properly explore their conformational space, observing multiple opening and closing events to conclude that 14‐3‐3‐ζ presents the highest opening propensity. To gain insight into this flexible behavior, we used correlation‐based tools to elucidate the mechanism of this process. A PCA analysis revealed that the dominant motions behind the observed flexibility of our systems resembled a bear trap mechanism, with fluctuating distances between helices H3 and H9 and a rigid behavior of H5 and H6, which acted as a hinge. Shortest path maps indicated that the most correlated residues during the MD simulations belong to a yet unknown small cavity at the outer side of the dimer, and opposite to the amphipathic groove, giving initial hints of an allosteric mechanism ruling the conformational plasticity of 14‐3‐3 proteins. We explain the higher opening rate of ζ by a distal interaction mediated by F153, a conserved residue between paralogs ζ, η, β, and τ located at the entrance of this small cavity. By a cross‐correlation analysis of distance time series obtained from MD simulations, we observed a high correlation between three events: an aliphatic–π interaction between the aromatic ring of F153 with the invariant A171—situated at the bottom of the cavity; the break of the H‐bond between D124 and N173; and the opening of the amphipathic groove. These results allowed us to propose a mechanism where the opening of the amphipathic groove is distally regulated by residues located at this small cavity formed by helices H6, H7, and H8. Interestingly, the X‐ray structure (PDB:4DNK) of the 14‐3‐3β paralog apo‐form shows one monomer in the closed and the other in the open conformation, where the open conformation exhibits the F153–A171 aliphatic–π interaction (distance 3.8 Å), the broken D124–N173 H‐bond, and an amphipathic groove opening at 25 Å, which is in excellent agreement with our simulations of open 14‐3‐3 forms. The H‐bond between these conserved residues and its implications on the flexibility of 14‐3‐3 have been previously reported for the paralog σ (Hu et al., 2014). We modeled single mutated paralogs and strengthened our hypothesis by observing more opening events for the γ_H157F mutant compared with its WT form. We continued exploring this mechanism and designed a third 14‐3‐3 mutant model by forcing the interaction between F153 and A171. To accomplish this, we mutated both residues to cysteines, defining a disulfide bond between them that resulted in a fully open 14‐3‐3 variant.

The modulation of 14‐3‐3's activity by binding inhibitors and enhancers has been studied for a long time. However, targeting the highly conserved 14‐3‐3's amphipathic groove to modulate protein–protein interactions would lead to unspecific multi‐paralog interactions. To cope with this, different strategies have already been proposed, including the design of compounds such as Fusicoccin‐A (Doveston et al., 2017) that bind to the interface generated between specific 14‐3‐3/phosphopeptide partners, or other small molecules interacting at secondary partner binding sites, located at the dimer interface (Valenti et al., 2019) or within a small hydrophobic pocket formed between helices H8 and H9 (Andlovic et al., 2024; Sijbesma et al., 2017). Our work indicates even different intervention strategies consisting of the regulation of 14‐3‐3 open/closed state plasticity. This may have far‐reaching consequences for target recognition and the stability of binding. In the previous in silico study of the 14‐3‐3/phosphopeptide complex formation, Hritz et al. showed that the open state of 14‐3‐3ζ is on the conformational pathway at both first contact and dissociation of the target (Nagy et al., 2017). Our in silico studies prove a new druggable cavity, paving the way for the development of novel therapeutic regulation of 14‐3‐3's via allosteric and paralog‐specific mechanisms.

## 4 MATERIALS AND METHODS

### 4.1 Molecular dynamics setup

MD simulations were performed with GROMACS 2021.5 (Abraham et al., 2015), using the Amber ff14SB force field (Maier et al., 2015) and the TIP3P water model (Jorgensen et al., 1983). The coordinates for paralogs γ, ζ, and ε were downloaded from the PDB, under the codes: 3UZD, 1QJA, and 2BR9, respectively. Target phospho‐peptides and crystallization solvents were removed, and missing loops from 3UZD and 1QJA were modeled with ModLoop (Fiser & Sali, 2003). Mutants γ_H157F, ζ_F153H, and ζ_F153C/A171C were generated by modifying the wild‐type PDB structures with Chimera (Pettersen et al., 2004). All 14‐3‐3 variants were centered in an octahedral simulation box and solvated, setting an ionic concentration of 150 mM using Na^+^ and Cl^−^. Systems were energy minimized using the steepest descent method; a 10 ns pressure (1 atm) and temperature (300 K) equilibration step was done using the Berendsen barostat and V‐rescale thermostat, restraining positions of backbone atoms. Simulations were held for 1 μs and ran by triplicate, starting from aleatory initial velocities. The Parrinello–Rahman barostat and Nose–Hoover thermostat were employed for production steps.

### 4.2 MD analyses

Inter‐residue distances were calculated with the VMD visualization software (Humphrey et al., 1996). SPMs were calculated with DynaComm (Casadevall et al., 2024), at the web server https://spmosuna.com/. To perform this analysis, mean distances and correlations between α carbons were calculated using the *cpptraj* module from AmberTools (Case et al., 2023). In this method, protein coordinates are simplified into a graph. Residues are represented by single nodes that are connected by edges when their distances are below a defined threshold and trajectory fraction. We evaluated distance thresholds of 6 and 8 Å and a trajectory fraction of 0.3. Edges are then weighted following Equation (1):

(1) $$l_{\mathrm{ij}}=-\log\left(\left|C_{\mathrm{ij}}\right|\right)$$

where *C*_*ij*_ corresponds to the correlation between *α* carbons and is calculated using Equation (2):

(2) $$C_{\mathrm{ij}}=\frac{\left\langle {\mathrm{\Delta r}}_{i}.{\mathrm{\Delta r}}_{j}\right\rangle }{\sqrt{\left\langle {\mathrm{\Delta r}}_{i}^{2}\right\rangle \left\langle {\mathrm{\Delta r}}_{j}^{2}\right\rangle }}$$

Δ*r*_*i*_ and Δ*r*_*j*_ are the displacements of the C*α* of the *i,j* residue using the X‐ray structure as a reference.

Finally, DynaComm applies the Dijkstra algorithm to identify residues having higher displacement correlations and therefore shorter edges, generating the shortest path to connect the first and last residues of the protein. For a detailed explanation of this algorithm, we refer the readers to the work of Csárdi and Nepusz (2006).

Cross‐correlation analysis between inter‐residue distances was done with Python's library *statsmodel*. Principal component analysis was done using different Gromacs analysis tools (Abraham et al., 2015). First, the covariance matrix of the peptide coordinate fluctuations was calculated with gmx covar. Then, with gmx anaeig, the trajectory was projected over the 1st and 2nd previously obtained eigenvectors, generating PC1 and PC2, and capturing the essential motions of the protein. For the last step, the tool gmx sham was employed to plot the Gibbs FEL. Briefly, the program generates a 2D histogram from these two variables (PC1 and PC2) and then performs a Boltzmann inversion of the histogram following Equation (3):

(3) $${\Delta \varepsilon }_{i}=-k_{B}T\left(\frac{n_{i}}{n_{\max}}\right)$$

where *k*_*B*_ is the Boltzmann constant, *T* is the simulated temperature (300 K), and *Δε*_*i*_ is the relative change in free energy between the conformations in the *i*th (*n*_*i*_) bin and the maximally occupied bin (*n*_*max*_). Cosine content was calculated with gmx energy. RMSD, RMSF, and secondary structure contents were also analyzed using GROMACS analysis tools: gmx rmsd, gmx rmsf, and gmx dssp, respectively.

Volumes of the small cavities were measured, and their druggabilities were estimated employing the VolSite method (Tran‐Nguyen et al., 2019), included in the OpenEye software package (Molecular Modeling Software, n.d.). Druggability scores are derived using a support vector machine model (Desaphy et al., 2012) containing multiple descriptors such as the cavity volume, a classification of regions within this volume according to different pharmacophoric types (hydrophobic, aromatic, hydrogen bond donor or acceptor, positive or negative ionizable, and null), and their accessibility. Cavities having positive scores are considered druggable.

### 4.3 Multiple sequence alignment

To analyze the inter‐species residue conservation of the identified 14‐3‐3 small cavity, multiple sequences of the 14‐3‐3 proteins were downloaded from the website www.ncbi.nlm.nih.gov and manually curated to avoid bias towards more represented/explored organisms (duplicate sequences were deleted). In addition, predicted, hypothetical, similar, and truncated sequences were deleted. At least 500 sequences corresponding to each of the canonical paralogs were independently locally aligned using Clustal Omega (Sievers & Higgins, 2018). After alignment, the resulting MSAs were visually analyzed using Protein Multiple Sequence Alignment Viewer (Sander et al., 2020), a fully client‐based software. After visual inspection of the MSA, those sequences that do not correspond to the 14‐3‐3 protein family were deleted, the sequences were realigned, and the MSA was visually inspected again. Finally, the amino acid frequency in the corresponding positions was analyzed using balcony R plugins (Płuciennik et al., 2018). The full form of the MSA can be downloaded at https://github.com/dbustoslab/MSA_protein_sci_2025.

## AUTHOR CONTRIBUTIONS

**Exequiel E. Barrera:** Conceptualization; formal analysis; funding acquisition; writing – original draft; writing – review and editing; investigation; visualization; methodology. **Rostislav Skrabana:** Investigation; funding acquisition; writing – review and editing; supervision. **Diego M. Bustos:** Conceptualization; formal analysis; investigation; methodology; visualization; supervision; writing – original draft; writing – review and editing.

## Supporting information


**FIGURE S1:** Surface representation of a 14‐3‐3 monomer showing inter‐paralog amino acid conservation on the BWR color scale from 0% to 100%. On the left, the view of the inner, highly conserved amphipathic groove, running parallel to the monomer base. On the right, a 180° rotation showing the much less conserved outer region of 14‐3‐3, where the small cavity is located.
**FIGURE S2:** Analysis of the cavity environment. (a) Multiple sequence alignment of the seven human paralog of 14‐3‐3 displaying the residues that form a small cavity at the opposite side of the amphipathic groove. (b) Surface representation for the ζ paralog showing such cavity, (c) an inset highlights their forming residues. Surface is colored with a radial distribution, using the geometrical center of the monomer and a GWR scale. Side chains in C are represented with balls and sticks and colored by atom name.
**FIGURE S3:** The cavity residues were analyzed for the amino acid conservation in the individual 14‐3‐3 isoforms. At least 500 sequences of each paralog were downloaded from Genbank and manually curated (predicted, hypothetical and truncated sequences were deleted). After curation, sequences were aligned with Clustal Omega (using default parameters) and each final MSA for the isoforms was analyzed using Balcony R plugins that generate a bar plot for each position in the alignment. The numbering corresponds to the ζ‐paralog, and the small number upon each bar corresponds to the percentage of those amino acids at that position.
**FIGURE S4:** Inter‐residue distances and RMSD time series of all simulated monomeric systems.
**FIGURE S5:** SPM analysis of each of the monomeric simulated systems, employing a distance threshold of 8 Å. Residues Asp and Asn involved in the hydrogen bond stabilizing closed conformations of the amphipathic groove are marked with dotted lines.
**FIGURE S6:** (a) Plot showing the eigenvalues of the top 10 eigenvectors calculated by PCA for each monomeric simulated system. (b) Cumulative variance of the top 20 eigenvectors for the same simulated systems.
**FIGURE S7:** Structural representation of the fluctuation between extreme conformations obtained by PCA. PC1 and PC2 are shown for every monomeric simulated system. In each of them 10 backbone representations are superimposed and colored with a BWR color scale, showing the main atomic displacements obtained from the 3 μs accumulated trajectories.
**FIGURE S8:** Root mean square fluctuation analysis indicating the degree of flexibility by residue. On the top of the plot a schematic representation of the secondary structure elements is shown. Residues forming each helix are the following: paralog‐ε (h1: 3–18; h2: 21–32; h3: 38–73; h4: 76–111; h5: 116–135; h6: 139–161; h7: 168–183; h8: 189–204; h9: 215–232). Paralog‐γ (h1: 3–18; h2: 20–33; h3: 38–71; h4: 78–108; h5: 118–136; h6: 141–163; h7: 170–185; h8: 190–206; h9: 216–234). Paralog‐ζ (h1: 3–16; h2: 19–32; h3: 38–64; h4: 77–108; h5: 113–131; h6: 138–159; h7: 165–181; h8: 186–204; 211–230).
**FIGURE S9:** Secondary structure analysis through the three simulated replicas for each monomeric system.
**FIGURE S10:** Analysis of dimeric 14‐3‐3ζ simulations. Cross‐correlation functions between distance time‐series of the residue pairs F153–A171 and N173–D124 (brown); and F153–A171 and G53–L220 (black).
**FIGURE S11:** Inter‐residue distances for the WT dimeric ζ‐paralog and its double F153C–A171C mutant. Results for each monomer are shown separately.
**TABLE S1:** Cosine content values of the first 5 eigenvectors calculated from the 3 μs accumulated trajectories of each simulated monomeric system.

## Data Availability

The data that support the findings of this study are available from the corresponding author upon reasonable request.
