# Supplementary material for: Deciphering opening mechanisms of 14‐3‐3 proteins
Source: Protein Sci. 2025 Mar 25;34(4):e70108. doi: 10.1002/pro.70108 (PMC11934215; doi:10.1002/pro.70108)
Supplement: Supplementary file 1 — FIGURE S1: Surface representation of a 14‐3‐3 monomer showing inter‐paralog amino acid conservation on the BWR color scale from 0% to 100%. On the left, the view of the inner, highly conserved amphipathic groove, running parallel to the monomer base. On the right, a 180° rotation showing the much less conserved outer region of 14‐3‐3, where the small cavity is located. FIGURE S2: Analysis of the cavity environment. (a) Multiple sequence alignment of the seven human paralog of 14‐3‐3 displaying the residues that form a small cavity at the opposite side of the amphipathic groove. (b) Surface representation for the ζ paralog showing such cavity, (c) an inset highlights their forming residues. Surface is colored with a radial distribution, using the geometrical center of the monomer and a GWR scale. Side chains in C are represented with balls and sticks and colored by atom name. FIGURE S3: The cavity residues were analyzed for the amino acid conservation in the individual 14‐3‐3 isoforms. At least 500 sequences of each paralog were downloaded from Genbank and manually curated (predicted, hypothetical and truncated sequences were deleted). After curation, sequences were aligned with Clustal Omega (using default parameters) and each final MSA for the isoforms was analyzed using Balcony R plugins that generate a bar plot for each position in the alignment. The numbering corresponds to the ζ‐paralog, and the small number upon each bar corresponds to the percentage of those amino acids at that position. FIGURE S4: Inter‐residue distances and RMSD time series of all simulated monomeric systems. FIGURE S5: SPM analysis of each of the monomeric simulated systems, employing a distance threshold of 8 Å. Residues Asp and Asn involved in the hydrogen bond stabilizing closed conformations of the amphipathic groove are marked with dotted lines. FIGURE S6: (a) Plot showing the eigenvalues of the top 10 eigenvectors calculated by PCA for each monomeric simulated system. (b) Cumulative [file PRO-34-e70108-s001.docx]

**Supplementary material from:**

DECIPHERING OPENING MECHANISMS OF 14-3-3 PROTEINS

Exequiel E. Barrera^1^, Rostislav Skrabana^2^ and Diego M. Bustos^1, 3^

1 Instituto de Histología y Embriología de Mendoza (IHEM), Universidad Nacional de Cuyo, CONICET, Mendoza, Argentina.

2 Institute of Neuroimmunology, Slovak Academy of Sciences, 845 10 Bratislava, Slovakia

3 Facultad de Ciencias Exactas y Naturales UNCUYO Mendoza Argentina.


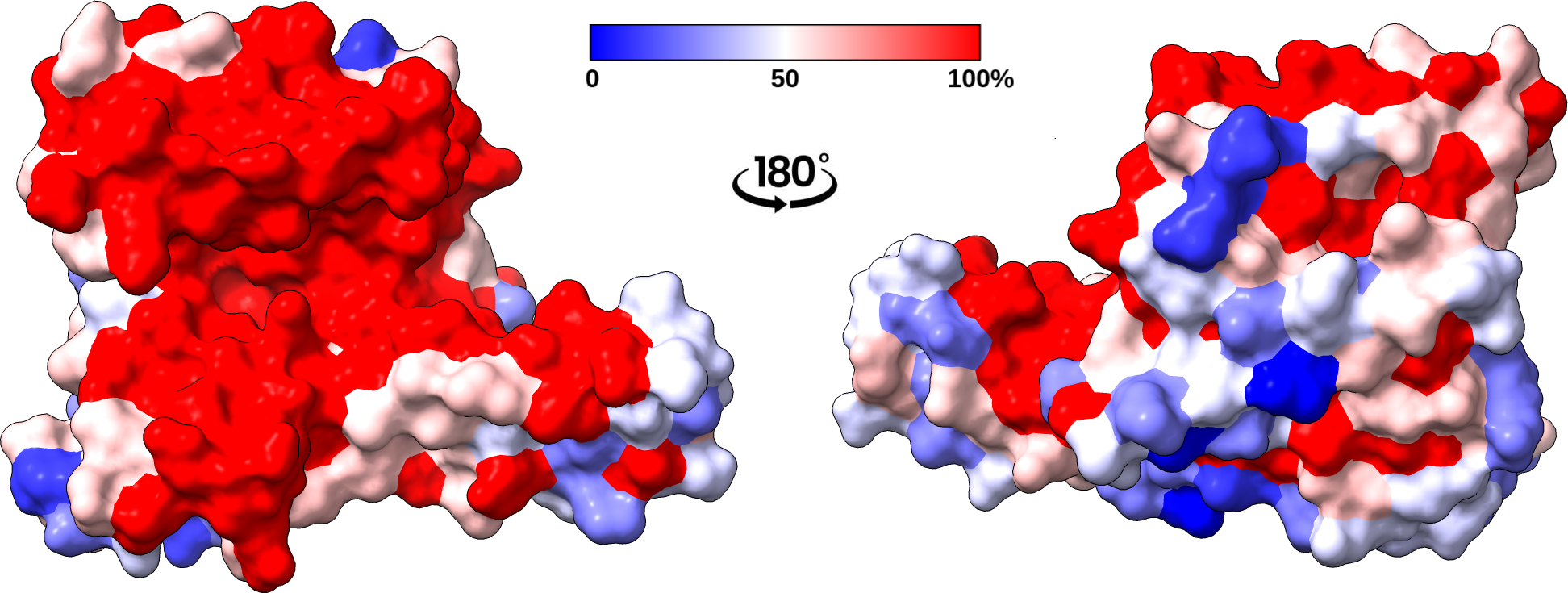


**Supplementary Figure 1.** Surface representation of a 14-3-3 monomer showing inter-paralog amino acid conservation on the BWR color scale from 0 % to 100 %. On the left, the view of the inner, highly conserved amphipathic groove, running parallel to the monomer base. On the right, a 180° rotation showing the much less conserved outer region of 14-3-3, where the small cavity is located.


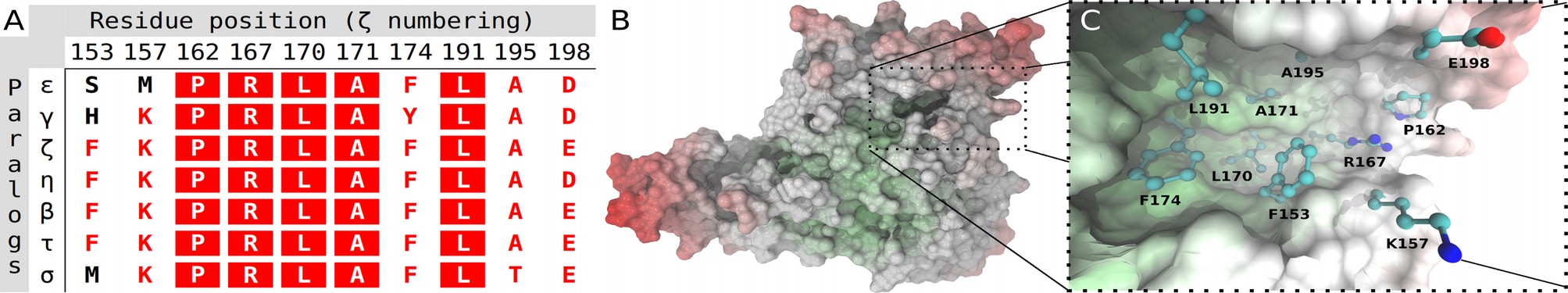


**Supplementary Figure 2.** Analysis of the cavity environment. (A) Multiple sequence alignment of the seven human paralog of 14-3-3 displaying the residues that form a small cavity at the opposite side of the amphipathic groove. (B) Surface representation for the ζ paralog showing such cavity, (C) an inset highlights their forming residues. Surface is colored with a radial distribution, using the geometrical center of the monomer and a GWR scale. Side chains in C are represented with balls and sticks and colored by atom name.


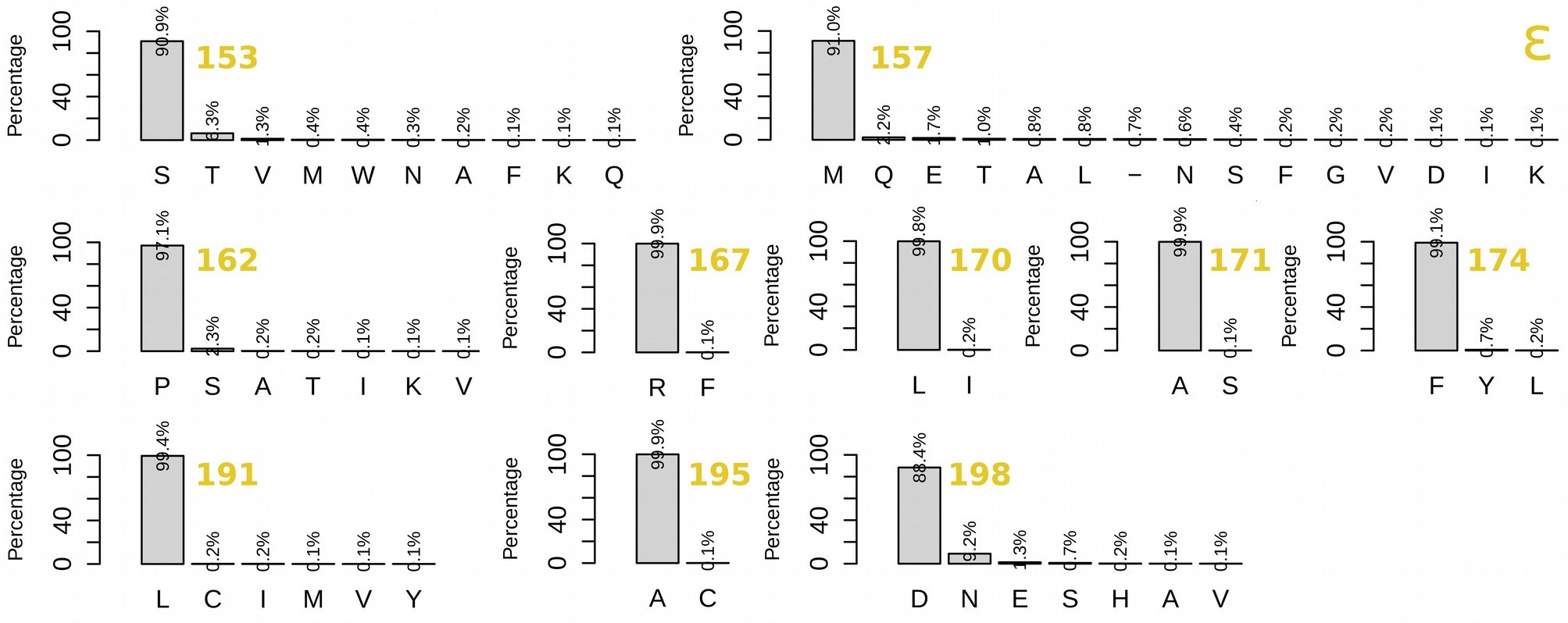


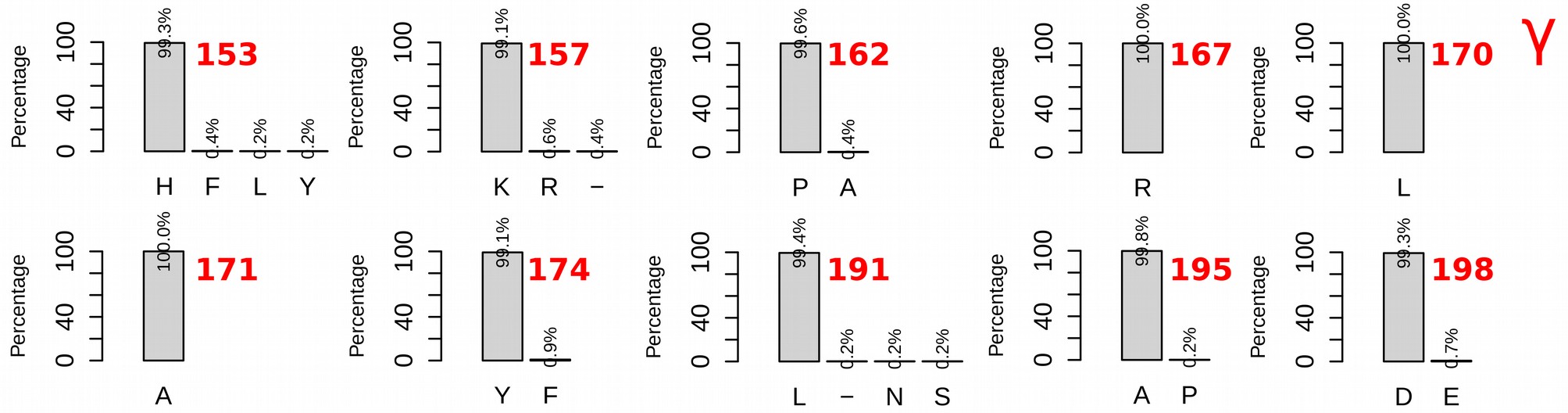


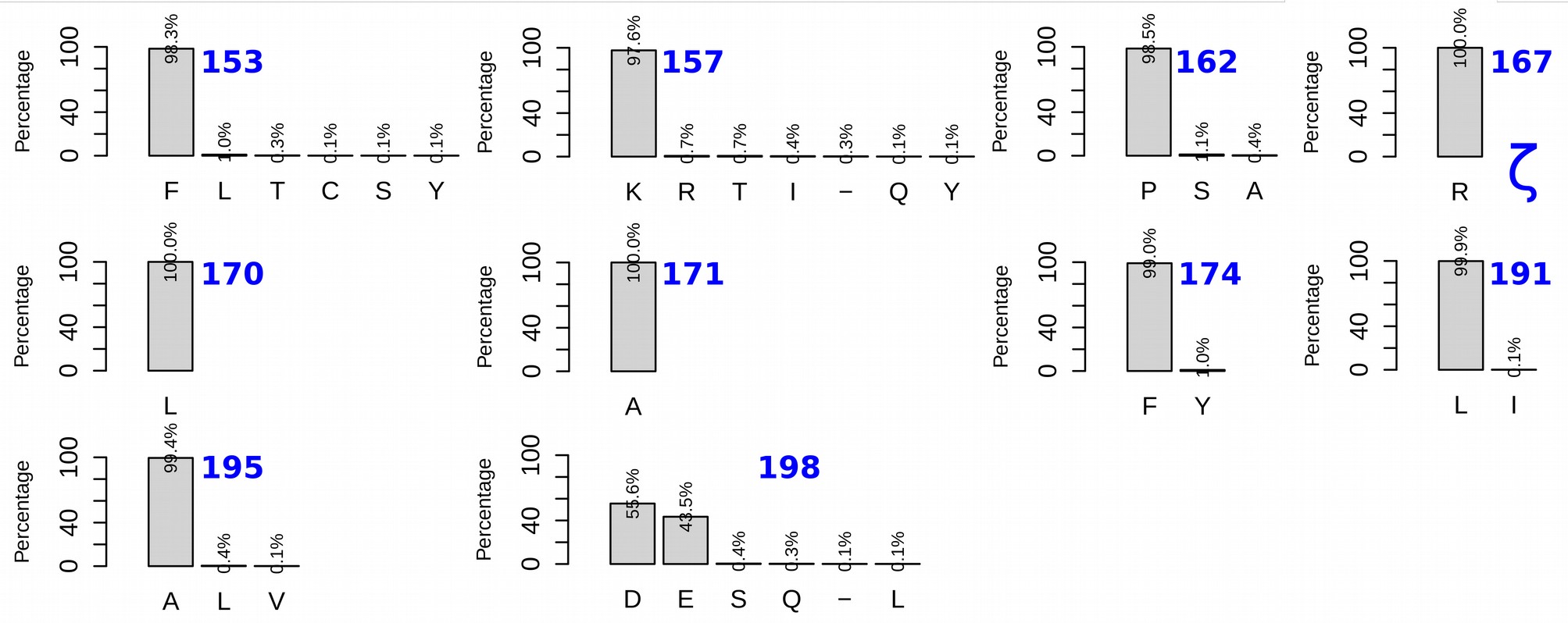


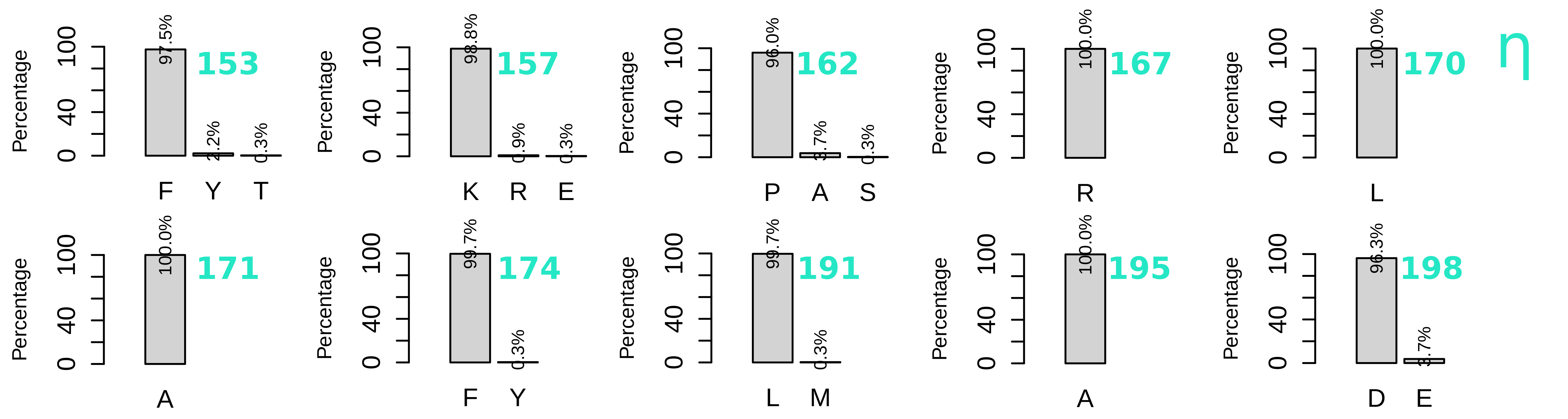


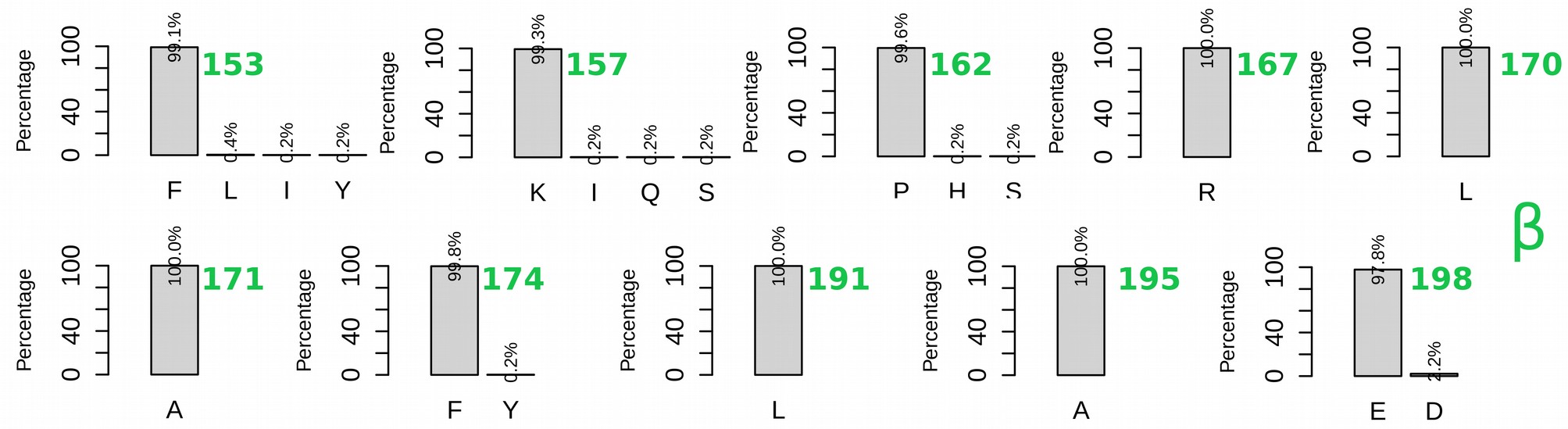


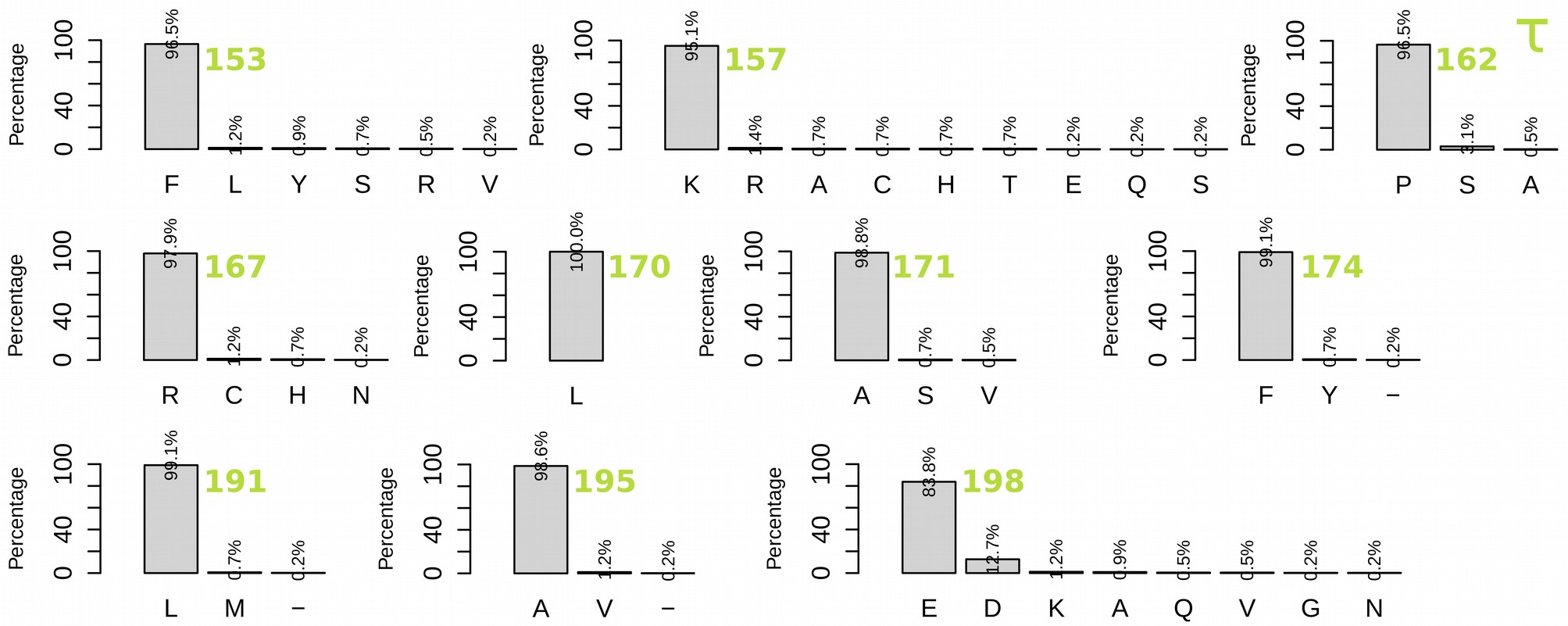


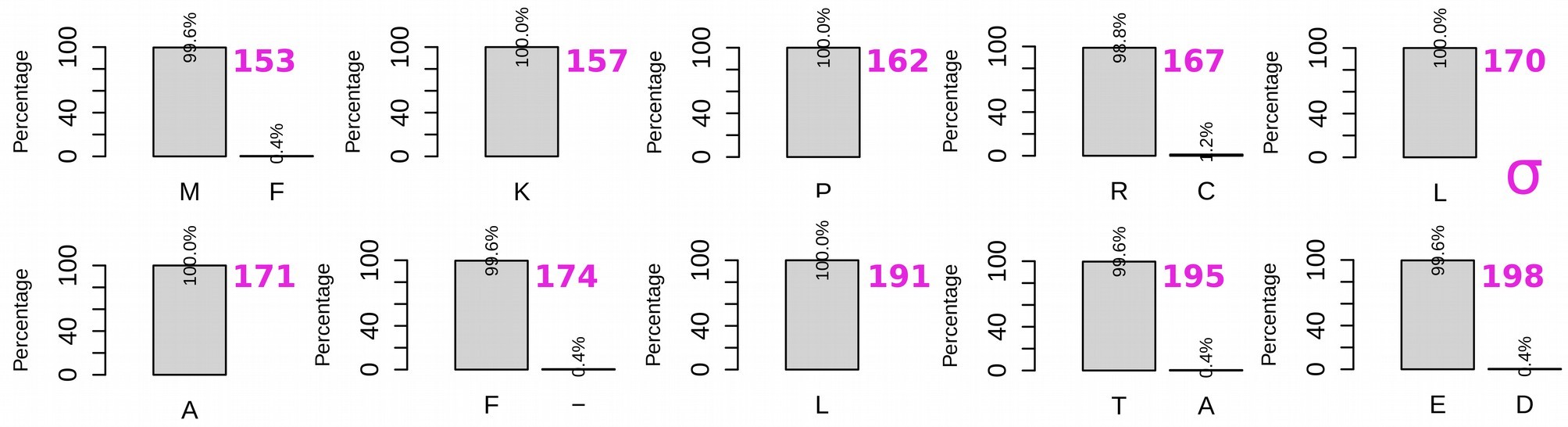


**Supplementary Figure 3.** The cavity residues were analyzed for the amino acid conservation in the individual 14-3-3 isoforms. At least 500 sequences of each paralog were downloaded from Genbank and manually curated (predicted, hypothetical and truncated sequences were deleted). After curation, sequences were aligned with Clustal Omega (using default parameters) and each final MSA for the isoforms was analyzed using Balcony R plugins that generate a bar plot for each position in the alignment. The numbering corresponds to the ζ-paralog, and the small number upon each bar corresponds to the percentage of those amino acids at that position

**
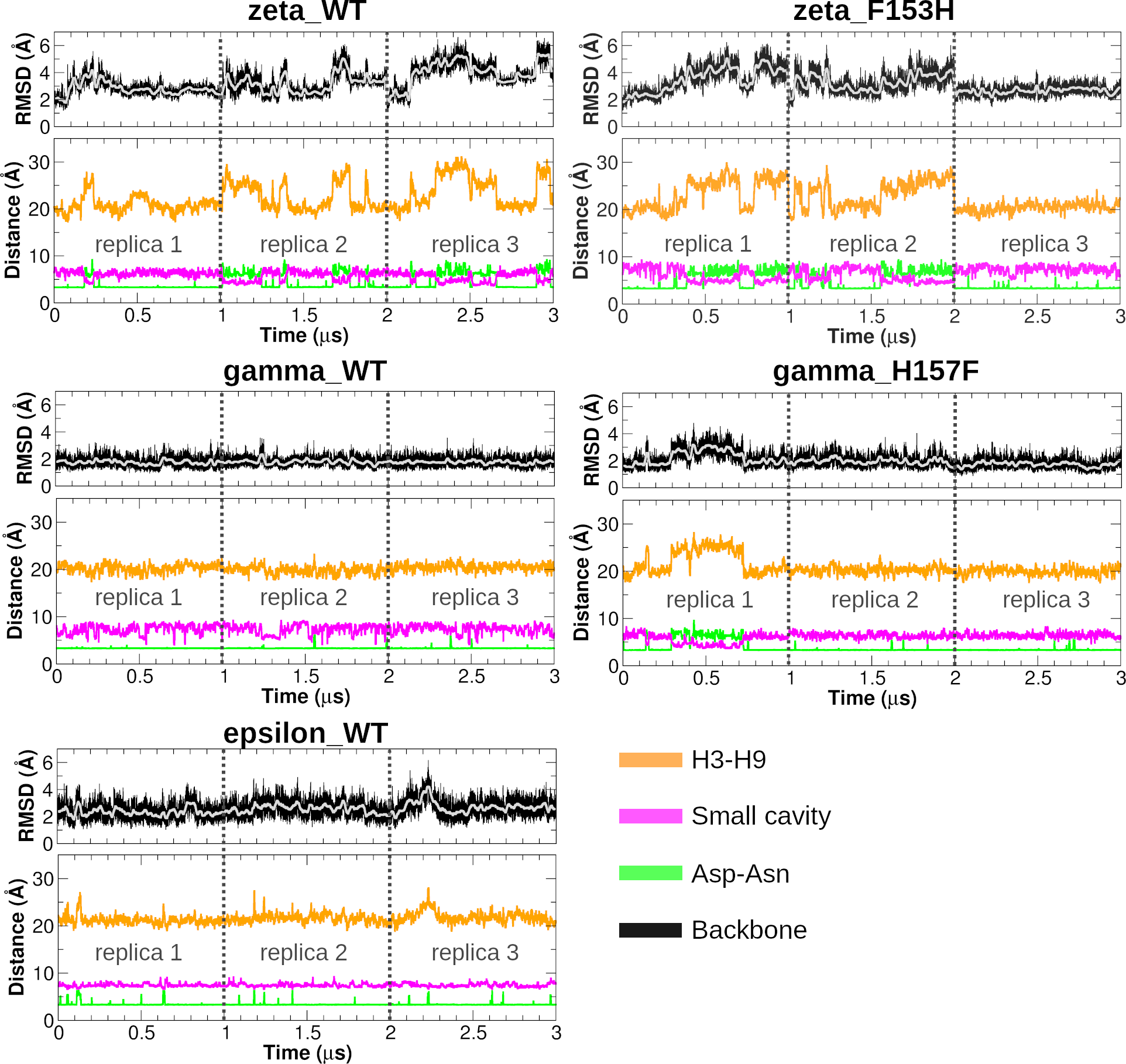
**

**Supplementary Figure 4.** Inter-residue distances and RMSD time series of all simulated monomeric systems.


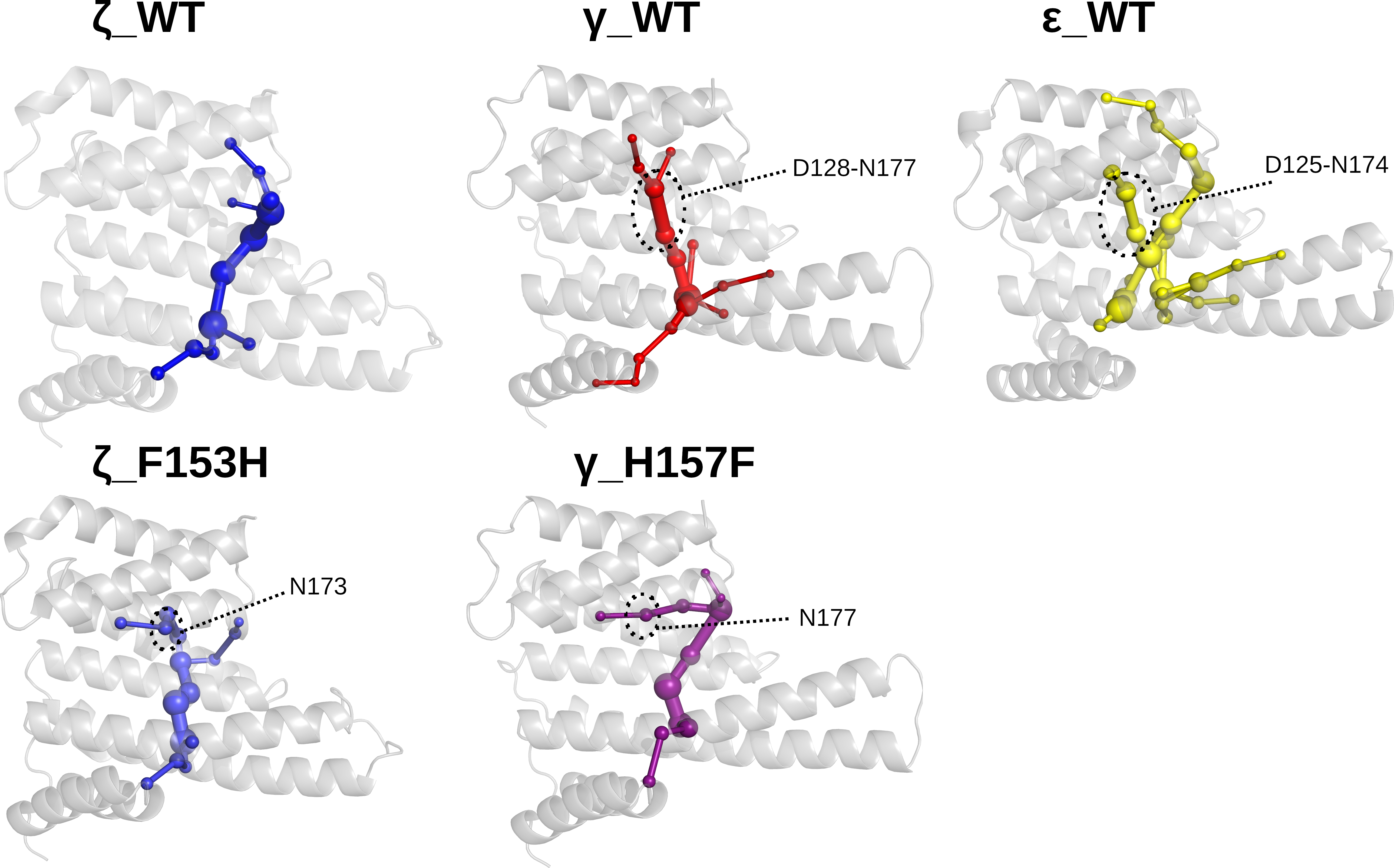


**Supplementary Figure 5.** SPM analysis of each of the monomeric simulated systems, employing a distance threshold of 8 Å. Residues Asp and Asn involved in the hydrogen bond stabilizing closed conformations of the amphipathic groove are marked with dotted lines.


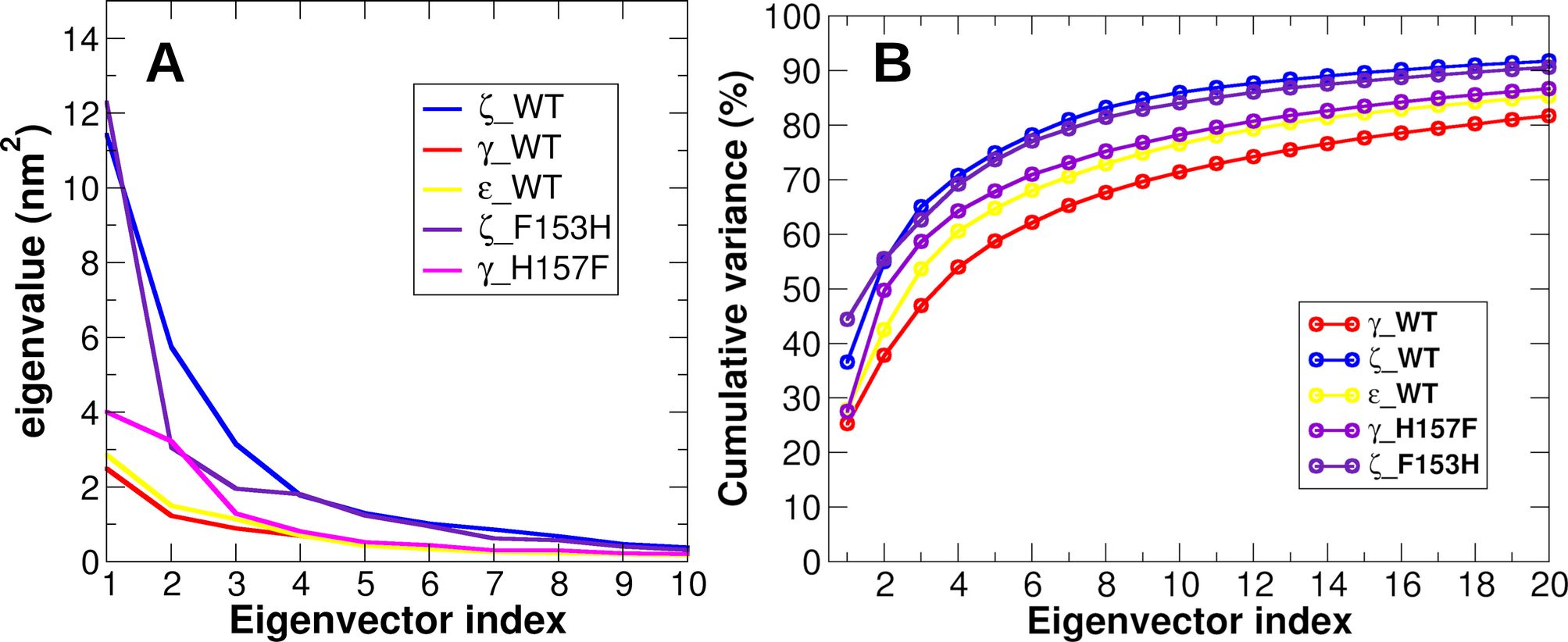


**Supplementary Figure 6.** A) Plot showing the eigenvalues of the top 10 eigenvectors calculated by PCA for each monomeric simulated system. B) Cumulative variance of the top 20 eigenvectors for the same simulated systems.


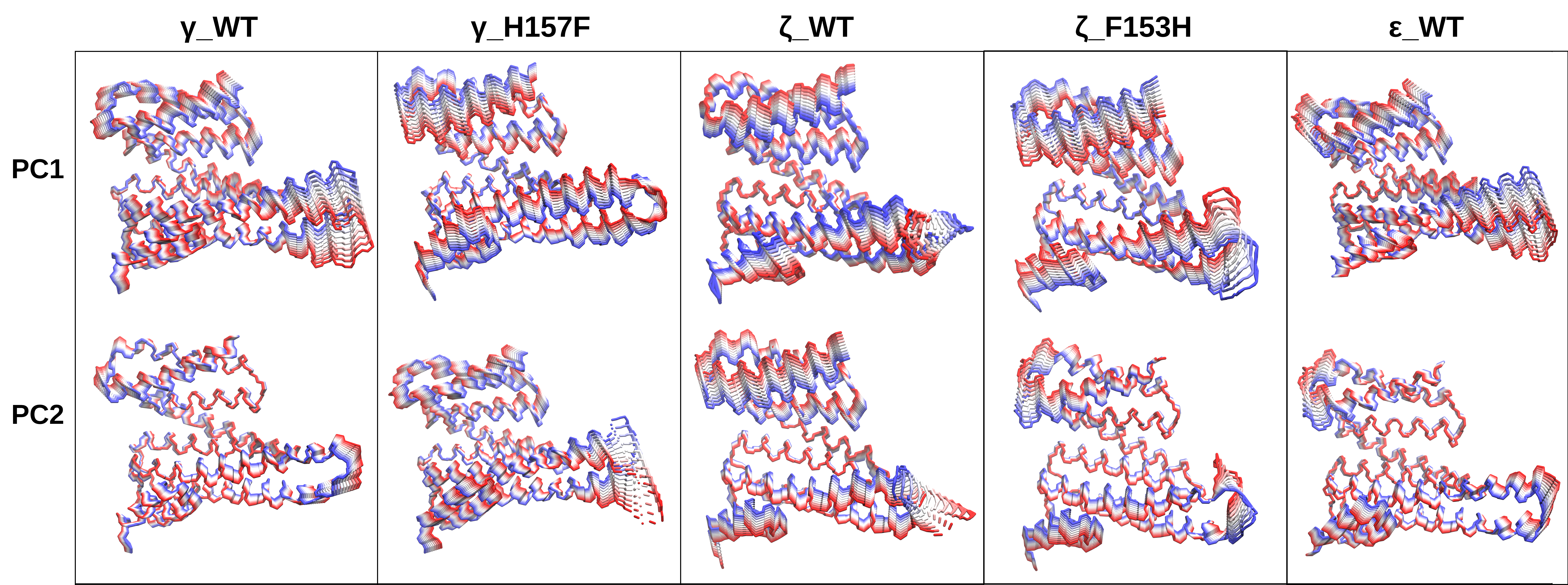


**Supplementary Figure 7.** Structural representation of the fluctuation between extreme conformations obtained by PCA. PC1 and PC2 are shown for every monomeric simulated system. In each of them 10 backbone representations are superimposed and colored with a BWR color scale, showing the main atomic displacements obtained from the 3 μs accumulated trajectories.


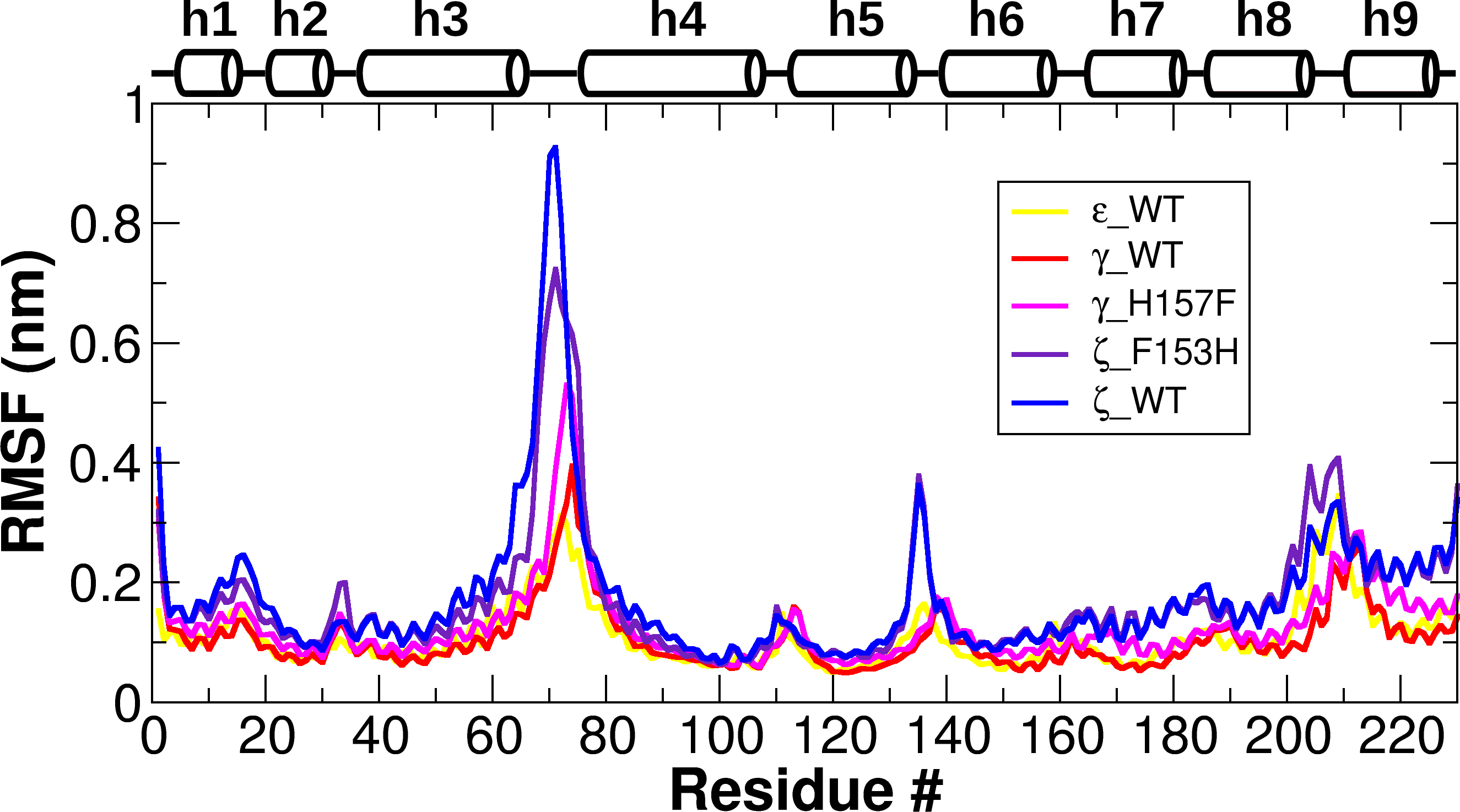


**Supplementary Figure 8.** Root mean square fluctuation analysis indicating the degree of flexibility by residue. On the top of the plot a schematic representation of the secondary structure elements is shown. Residues forming each helix are the following: paralog-ε (h1: 3-18; h2: 21-32; h3: 38-73; h4: 76-111; h5: 116-135; h6: 139-161; h7: 168-183; h8: 189-204; h9: 215-232). Paralog-γ (h1: 3-18; h2: 20-33; h3: 38-71; h4: 78-108; h5: 118-136; h6: 141-163; h7: 170-185; h8: 190-206; h9: 216-234). Paralog-ζ (h1: 3-16; h2: 19-32; h3: 38-64; h4: 77-108; h5: 113-131; h6: 138-159; h7: 165-181; h8: 186-204; 211-230)


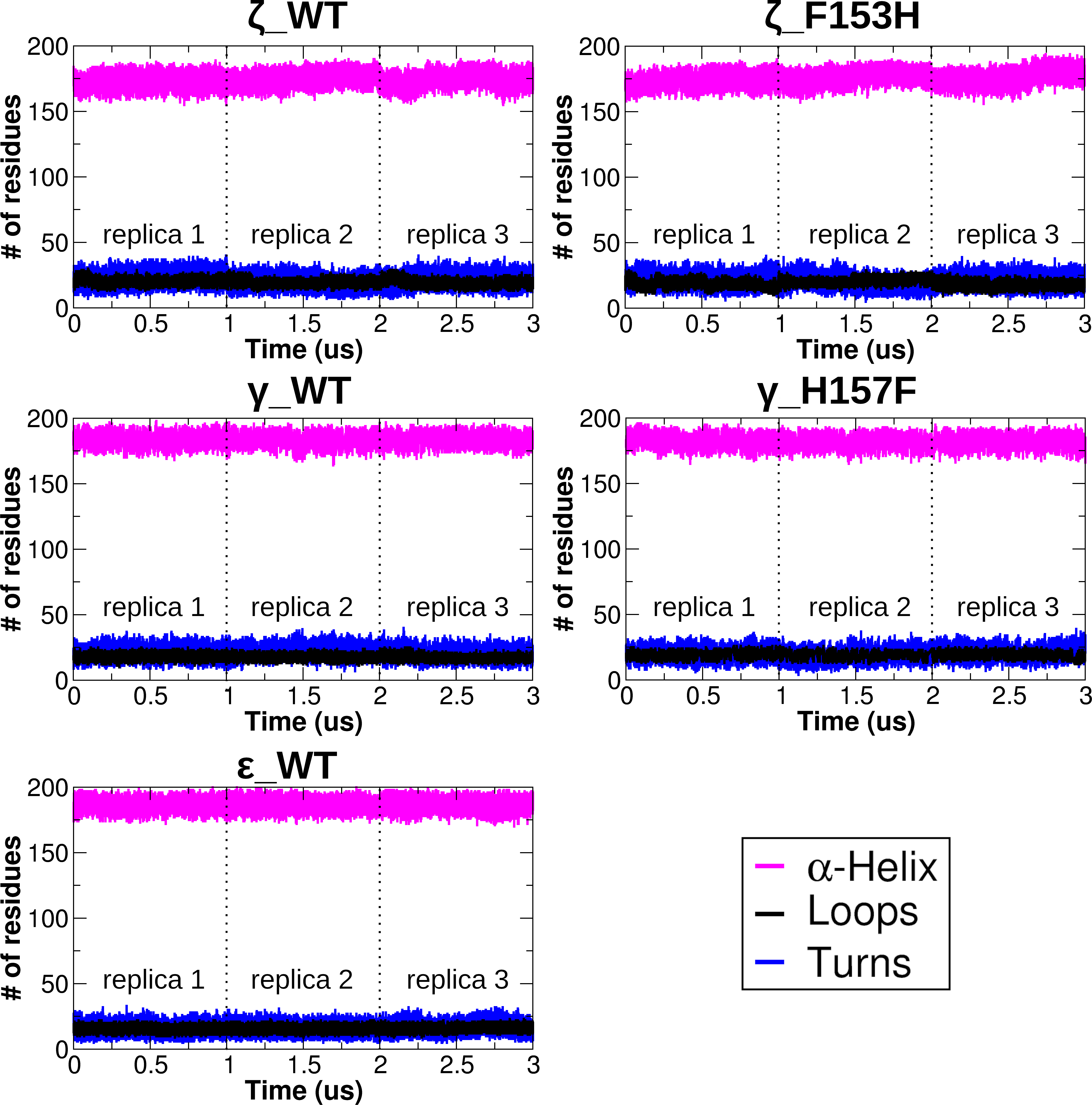


**Supplementary Figure 9.** Secondary structure analysis through the 3 simulated replicas for each monomeric system.


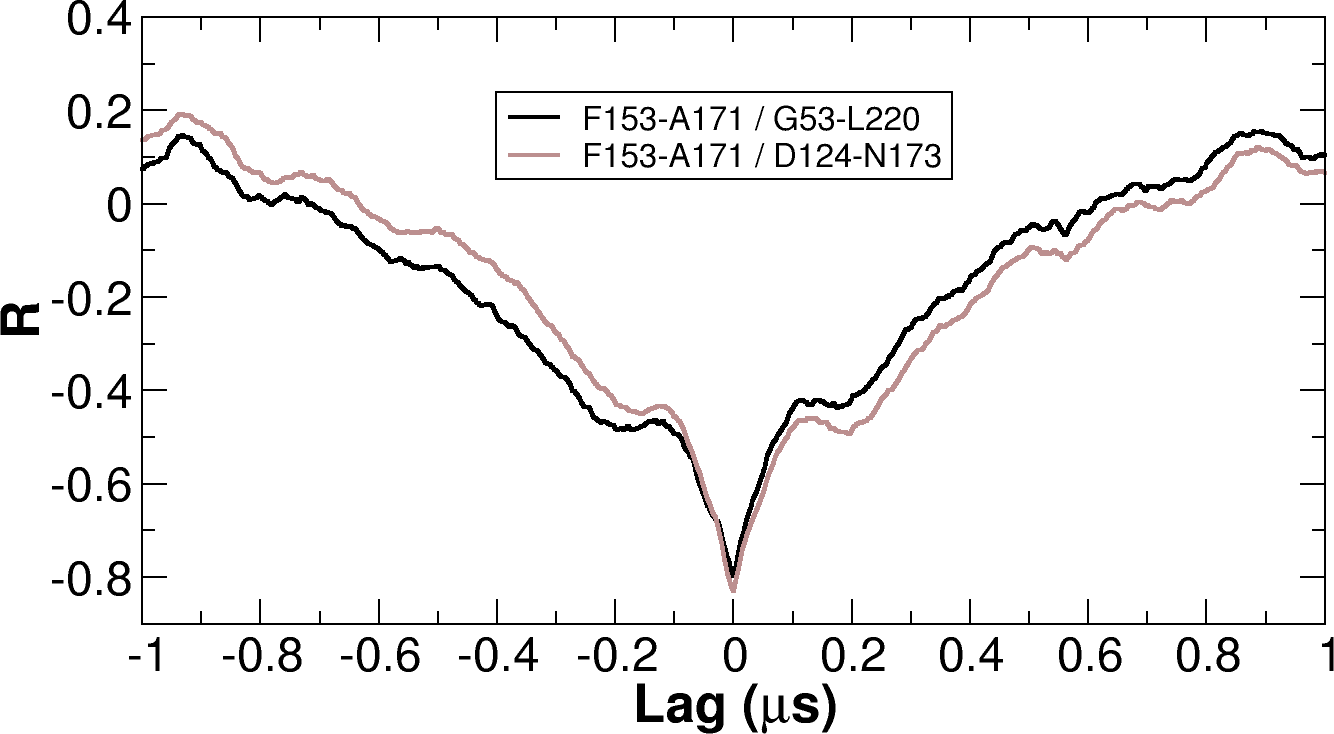


**Supplementary Figure 10.** Analysis of dimeric 14-3-3ζ simulations. Cross-correlation functions between distance time-series of the residue pairs F153-A171 and N173-D124 (brown); and F153-A171 and G53-L220 (black).


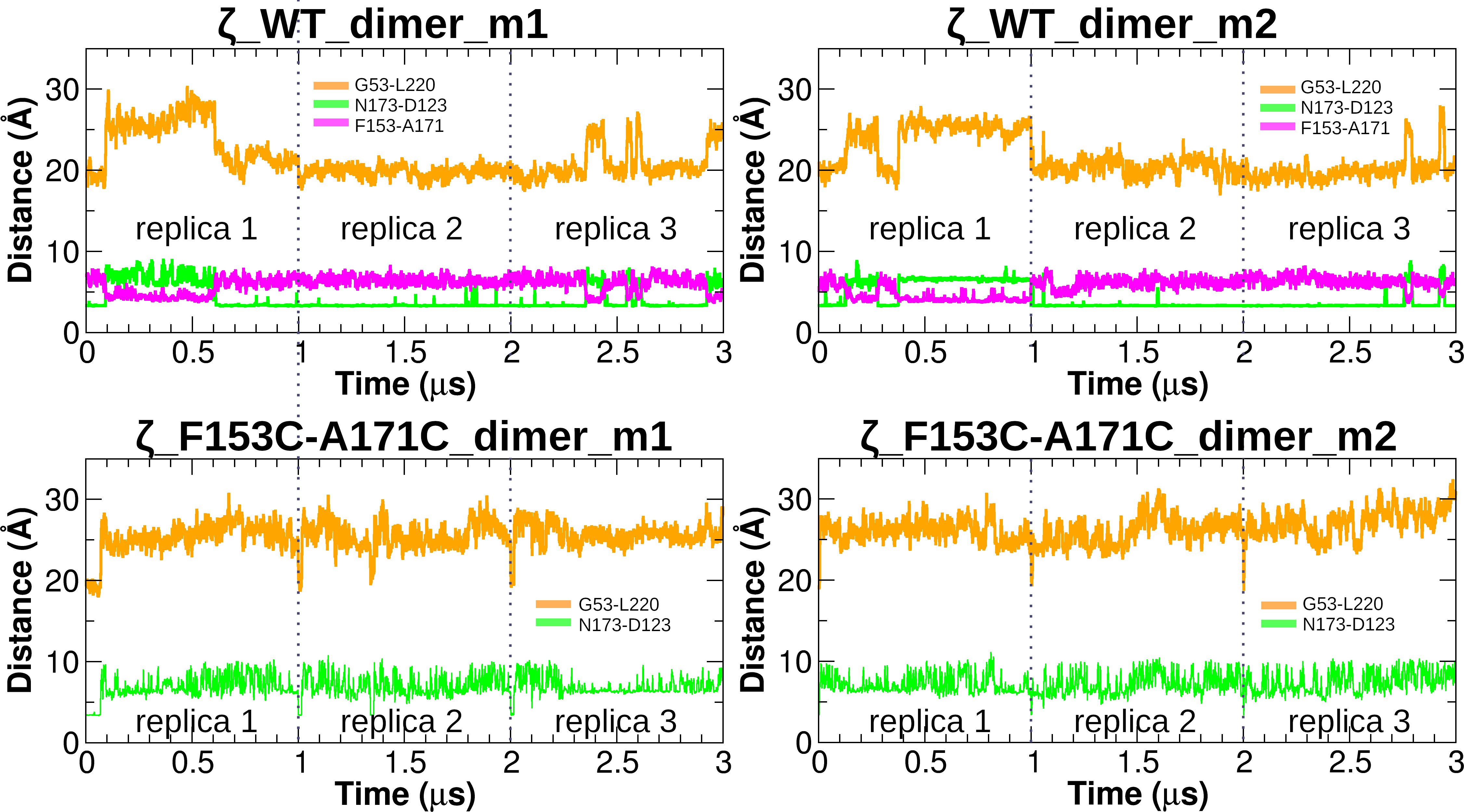

**Supplementary Figure 11.** Inter-residue distances for the WT dimeric ζ-paralog and its double F153C-A171C mutant. Results for each monomer are shown separately.

|  | pc1 | pc2 | pc3 | pc4 | pc5 |
| --- | --- | --- | --- | --- | --- |
| ζ_WT | 0.45967 | 0.09615 | 0.01796 | 0.00063 | 0.07566 |
| γ_WT | 0.00013 | 0.00869 | 0.00670 | 0.01865 | 0.01268 |
| ε_WT | 0.05968 | 0.02613 | 0.04288 | 0.00034 | 0.00048 |
| ζ_F153H | 0.13370 | 0.00001 | 0.05215 | 0.05680 | 0.12549 |
| γ_H157F | 0.19247 | 0.00007 | 0.01648 | 0.00050 | 0.02448 |

**Supplementary Table 1.** Cosine content values of the first 5 eigenvectors calculated from the 3 μs accumulated trajectories of each simulated monomeric system.
